# Leaf-based energy harvesting and storage utilizing hygroscopic iron hydrogel for continuous power generation

**DOI:** 10.1038/s41467-025-60341-z

**Published:** 2025-06-06

**Authors:** Shuai Guo, Yaoxin Zhang, Zhen Yu, Ming Dai, Xuanchen Liu, Hongbo Wang, Siqi Liu, J. Justin Koh, Wanxin Sun, Yuanping Feng, Yuanzheng Chen, Lin Yang, Peng Sun, Geyu Lu, Cunjiang Yu, Wenshuai Chen, Stefaan De Wolf, Zuankai Wang, Swee Ching Tan

**Affiliations:** 1 Department of Materials Science and Engineering, 9 Engineering Drive 1, Singapore, 117575 Singapore; 2 China-UK Low Carbon College, Shanghai Jiao Tong University, Shanghai, 201306 P.R. China; 3 Key Laboratory of Bio-based Material Science and Technology, Ministry of Education, Northeast Forestry University, Harbin, 150040 P.R. China; 4 Department of Mechanical Engineering, The Hong Kong Polytechnic University, Hong Kong, P.R. China; 5 Institute of Materials Research and Engineering (IMRE), Agency for Science, Technology and Research (A*STAR), 2 Fusionopolis Way, Innovis #08-03, Singapore, 138634 Singapore; 6 Division of Nano Surfaces, Bruker Corporation, 11 Biopolis Way, Singapore, 138667 Singapore; 7 Department of Physics, National University of Singapore, 2 Science Drive 3, Singapore, 117551 Singapore; 8 School of Physical Science and Technology, Key Laboratory of Advanced Technologies of Materials, Southwest Jiaotong University, Chengdu, 610031 P.R. China; 9 Key Laboratory of Optoelectronic Technology and System of Ministry of Education, College of Optoelectronic Engineering, Chongqing University, Chongqing, 400044 P. R. China; 10 State Key Laboratory on Integrated Optoelectronics, College of Electronic Science and Engineering, Jilin University, Changchun, China; 11 Department of Electrical and Computer Engineering, University of Illinois Urbana-Champaign, Urbana, IL 61801 USA; 12 Department of Materials Science and Engineering, University of Illinois, Urbana-Champaign, Urbana, IL USA; 13 Department of Mechanical Science and Engineering, University of Illinois, Urbana-Champaign, Urbana, IL USA; 14 Department of Bioengineering, Materials Research Laboratory, Beckman Institute for Advanced Science and Technology, Nick Holonyak Micro and Nanotechnology Laboratory, University of Illinois, Urbana-Champaign, Urbana, IL USA; 15 Physical and Engineering Division (PSE), King Abdullah University of Science and Technology (KAUST), Thuwal, 23955-6900 Kingdom of Saudi Arabia

**Keywords:** Chemical engineering, Energy harvesting, Energy storage, Materials for energy and catalysis

## Abstract

In the era of big data, developing next-generation self-powered continuous energy harvesting systems is of great importance. Taking advantage of fallen leaves’ specific structural advantage gifted by nature, we propose a facile approach to convert fallen leaves into energy harvesters from ubiquitous moisture, based on surface treatments and asymmetric coating of hygroscopic iron hydrogels. Upon moisture absorption, a water gradient is established between areas with/without hydrogel coating, and maintained due to gel-like behaviors and leaf veins for water retention and diffusion restriction, thus forming electrical double layers over the leaf surface and showing capacitance-like behavior for energy charging and discharging. Besides, the specific leaf cell structures with small grooves enabled uniform carbon coatings instead of aggregations, and high electrical conductivity, resulting in 49 μA/cm^2^ and 497 μW/cm^3^ electrical output, achieving competitive performance with the state-of-art and potential for lower environmental impact compared to other types of energy harvesters.

## Abstract (web summary)

In this work, authors convert fallen leaves into energy harvesters using hygroscopic iron hydrogel, achieving continuous power generation from moisture. The device delivers high current density and power output with potential for lower environmental impact compared to alternative harvesters.

## Introduction

In the era of big data and the Internet of Things (IoTs), developing renewable energy for self-powered wearable technologies is of great urgency^1–5^. The emergence and fast booming of triboelectric nanogenerators (TENGs), which rely on contact-separation, provide an effective approach to harvesting motion energy, and have been applied in many application scenarios, including self-powered implantable patches^6^, electrotherapy^7^, and microbial disinfections^8–10^. However, external motion is required for TENG, which may pose some restrictions.

Atmospheric moisture is emerging as a promising alternative renewable energy source since the ubiquity of moisture on earth could overcome the restrictions from other renewable energy technologies^11–14^. The last five years have witnessed a surge of interest in developing hygroscopic materials for moisture capture, including hydrogels, metal-organic frameworks, and composite materials, which provide an important prerequisite for lateral energy conversion^15–28^. However, how to effectively harvest such a tremendous amount of moisture in a sustainable manner, including materials for moisture sorption, environmental friendly processing methods, and cost-efficient device assembly techniques is a long-standing problem for harvesting energy from moisture, especially when considering the limited power output from the current stage self-powered devices^29–32^. Hence, a combination of materials with their specific structures and a sustainable fabrication process would greatly facilitate the overall sustainability and power density of energy harvesting that potentially allows them in real-world application scenarios.

The delicate structures of leaves gifted by nature could be a promising choice for water and energy harvesting^33,34^. For instance, their surfaces with nano-micro cell structures, known as Lotus Leaves Effects, provide a specific hydrophobic self-cleaning surface, which is a valuable design principle for nature-inspired lubricant coating and droplet-repellent water and energy harvesters^35–38^. The exclusive light harvesting and photosynthesis process of leaves, realized by photosystem II in mesophyll cells, are either directly applied in bio-based solar cells and photovoltaic devices or mimic its energy conversion process for leaf-inspired artificial photosynthesis^39–41^. Compared to the leaf cells, the leaf veins, which play an important role in transporting water and nutrients to mesophyll for photosynthesis, endow improved mechanical strength due to the high concentration of lignin and hierarchical porous structures, inspiring the additive manufacturing of fiber-reinforced composites^42–45^. In addition, the biodegradability of leaves also makes them suitable as templates or raw materials for developing sustainable, biodegradable products, such as packaging materials and composites^46,47^.

**Fig. 1:**
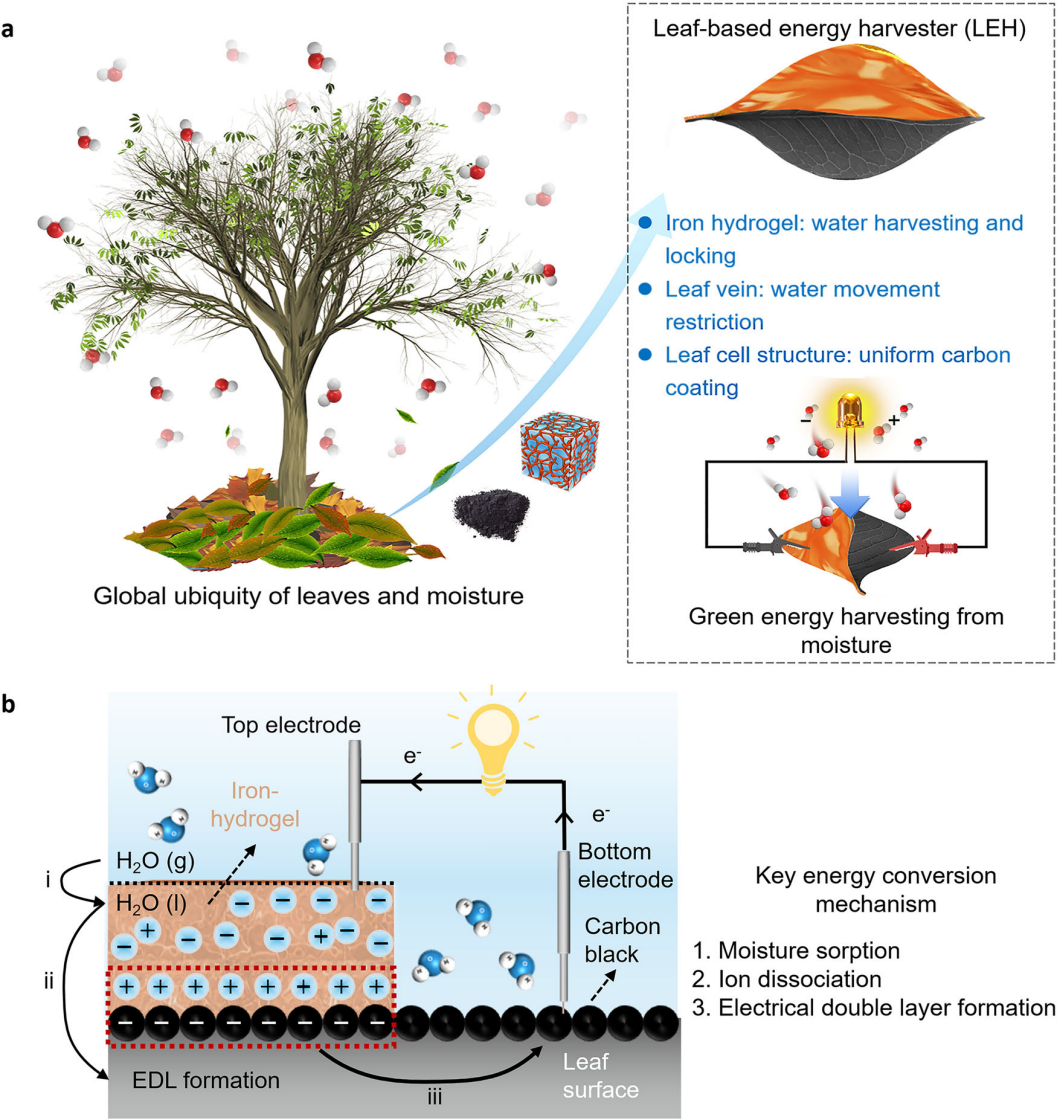
Converting fallen leaves into leaf-based energy harvesters (LEHs) from ambient moisture. **a** Schematic illustration of the advantages of converting fallen leaves into self-powered energy harvesters from ambient moisture. The specific leaf cell groove structures, leaf central veins, and hygroscopic hydrogel with network structures play a significant role in improving and prolonging energy harvesting performance. **b** The energy generation mechanism of LEH. A phase change of water from the gaseous state to the liquid state could trigger the dislocation of iron hydrogel (i). Carbon black surface becomes negatively charged after contact with liquid water, followed by the electrical double layer (EDL) formation through cation adsorption (ii). Under external circuits, the electrons transfer to the external electrical appliances for power generation (iii).

Taking advantage of the combined leaves’ distinct merits and reaching the aim of developing renewable energy, herein, we propose a facile route to convert fallen leaves into a leaf-based energy harvester (LEH) by utilizing an asymmetric structure with a super-hygroscopic iron hydrogel. LEHs are readily fabricated through a conductive treatment of delignified leaves by dip-coating them in carbon black (CB), followed by coating with a hygroscopic iron hydrogel on one side of the leaf surface. Owing to the gel-like structures and the leaf veins, the captured moisture could be locked inside the iron hydrogel, and the water movement to another dry side is cut off by the vein (Fig. 1a). Thus, a long-lasting water gradient across LEHs can be quickly formed, inducing a built-in potential difference (~0.5 V, >200 h) upon hydrogel absorption of moisture from the air. A systematic study of the mechanism reveals that the hygro-contact-ionic interaction between the hydrogel and the negatively charged CB surface on the LEH leads to the formation of electrical double layers (EDLs), and the energy scavenged by the LEH is stored inside these EDLs. Once the external circuits are applied, these EDLs could be discharged, which could be quickly replenished within 1/30 of discharging time by either external resources or ubiquitous moisture. Such a self-regeneration process guarantees continuous power output (Fig. 1b). In addition, fallen leaves could be considered an ideal substrate for moisture energy harvesting, due to their specific groove cell structures and thickness, allowing CB to readily arrange on their surface, resulting in the highest electrical conductivity compared with other commonly used substrates. As a result of these favorable properties, LEHs could deliver a short circuit current density (*I*_sc_) and volumetric power density as high as 49 μA/cm^2^ and 497 μW/cm^3^, respectively. Life cycle assessment indicates that the environmental impacts of LEHs are much lower than other categories of energy harvesters. With further consideration of the large abundance of raw materials (Fig. 1a, over 8,800,000 tons in 2018 in the United States alone, equivalent to 1.76 billion filled rubbish bags^48,49^), the LEH exhibits a combination of high-power output, environmental benignity, and low cost, which outperforms the vast majority of self-powered energy harvesters with different substrates (Supplementary Fig. 1, evaluation standard in Supplementary Note 1). Finally, a power panel through the integration of LEHs is readily developed with ~13 V voltage output and ~0.2 mA/cm^2^ current output for powering electronic devices^29^.

## Results

### Moisture sorption of super-hygroscopic iron hydrogel

**Fig. 2:**
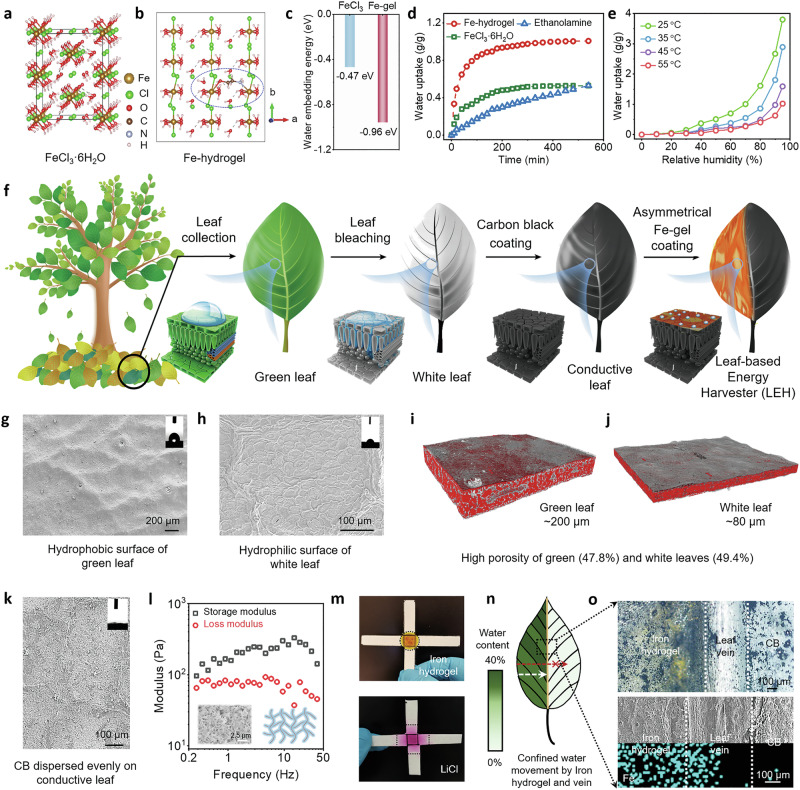
Characterization of iron hydrogel and LEH. **a** DFT stimulation of an optimized FeCl_3_·6H_2_O unit cell. **b** Optimized (100) slab configuration for water molecule absorption of a 1 × 2 × 1 iron hydrogel supercell. The brown, green, red, black, silver, and white spheres represent Fe, Cl, O, C, N, and H atoms, respectively. **c** Water embedding energy of FeCl_3_·6H_2_O and the iron hydrogel. The more negative value for the iron hydrogel indicates a stronger water affinity. **d** Water uptake performance of the iron hydrogel, FeCl_3_·6H_2_O and EA under 25 °C, 75% RH. The iron hydrogel exhibited both higher moisture uptake capacity and faster water capture kinetics. **e** Moisture absorption isotherm of iron hydrogel under different temperatures. **f** Schematic illustration of the fabrication process of LEH. The preparation process could be summarized into fallen leaves collection, leaf bleaching, conductive treatment, and asymmetrical iron hydrogel coating. **g**, **h** SEM images and water contact angle measurements of (**g**) a pristine green leaf and (**h**) a white leaf. An inversion of surface wettability was observed after surface bleaching. **i**, **j** X-ray CT image of (**i**) a pristine green leaf and (**j**) a white leaf, indicating the high porosity of the leaf. **k** SEM image and water contact angle of a conductive leaf. **l** Rheological properties of iron hydrogel under hydrate conditions. The storage modulus of the iron hydrogel is higher than the loss modulus, implying a gel-like behavior for water movement restriction. Inset: SEM image of the iron hydrogel with a network structure. **m** The directional water diffusion images of the iron hydrogel and LiCl after 24 h of moisture absorption at 75% RH. Both solutions are evenly dip-coated in the central square area drawn by the solid line and dried in the oven. **n** Water content distribution of LEH after moisture absorption. An apparent water gradient across LEH was observed. **o** Optical, SEM images, and EDS mapping of the LEH. A clear leaf vein is observed, acting as a barrier between the iron hydrogel and the untreated area after asymmetrical coating.

The iron hydrogel was synthesized by a coordination reaction between iron chloride hexahydrate (FeCl_3_·6H_2_O) precursor and ethanolamine (EA) ligand. This reaction could also be deemed as an acid-base reaction, in which FeCl_3_·6H_2_O acts as a Lewis acid and electron acceptor, while EA acts as a Lewis base and electron donor. To further characterize the specific structure of the iron hydrogel, especially the identification of the coordination sites, first-principle calculations were carried out using density-functional theory (DFT, Supplementary Note 2). As depicted in Fig. 2a, DFT simulations started from the optimization of FeCl_3_·6H_2_O unit cells, followed by embedding one EA molecule into the optimized unit cells to mimic iron hydrogel formation. As shown in Fig. 2b, both nitrogen and oxygen atoms in EA could coordinate with iron atoms in the dissected (110) slab configuration. Fourier-transform infrared spectroscopy (FT-IR), X-ray photoelectron spectroscopy (XPS), and scanning electron microscopy (SEM) were then applied to further confirm such coordination interactions (Supplementary Figs. 2–5, Supplementary Note 3)^50–53^. A network structure with much lower water embedding energy compared with the precursor was observed (Fig. 2c), implying a much higher water uptake capacity and faster moisture capture kinetics. This speculation was supported by experimental tests. The iron hydrogel quickly reached a plateau of 0.92 g/g water uptake within 180 min, however, only 0.47 g/g and 0.28 g/g water uptake of FeCl_3_·6H_2_O and EA could be achieved under the same conditions (Fig. 2d and Supplementary Figs. 6,7). No significant reduction in moisture uptake capacity was found after 50 absorption/desorption cycles, implying the superior stability and reusability of the iron hydrogel (Supplementary Fig. 8). According to the moisture absorption isotherms in Fig. 2e, the iron hydrogel could harvest 3.79 g/g moisture under room temperature. Considerable moisture uptake could also be realized at 35 °C, demonstrating a wide LEH working temperature range. Only minimal moisture uptake could be detected at 55 °C, implying that the desorption of iron hydrogel could take place above this temperature. Thermogravimetric analysis (TGA) was then carried out to determine the desorption temperature. Due to the network structure of the iron hydrogel, most of the absorbed moisture was released at 70 °C, while less than 50% of the water was liberated from FeCl_3_·6H_2_O (Supplementary Fig. 9). Lower regeneration temperature is also beneficial for sustainable reuse of LEHs since less energy input is required for LEH regeneration.

### LEH fabrication and characterization

As shown in Fig. 2f, LEHs were readily fabricated through the collection of fallen leaves, bleaching, conductive treatment, and asymmetrical coating of the prepared iron hydrogel. The hydrophobic nature of the pristine leaf would hinder further conductive treatment and iron hydrogel coating, so surface bleaching was carried out first. The reduction of the water contact angle from 105° to 60° together with the enhanced intensity of C=O (1656 cm^−1^) and C-O (1236 cm^−1^) bonds in the FT-IR spectra and increased oxygen content as measured by XPS demonstrated the change in surface wettability after the bleaching treatment (Supplementary Figs. 10, 11)^54^. Besides, leaf cells were more clearly exposed after decolouring compared with that of the pristine leaf (Fig. 2g, h). Furthermore, the inherently high porosity (>47% porosity) of leaves was another important reason for its selection as the substrate material since it could be readily impregnated with CB to build a conductive network (Fig. 2i, j). A reduction in leaf thickness from 200 to 80 μm was observed. After conductive treatment with CB, a homogenous distribution of CB particles was observed over the leaf substrate, and the surface became super hydrophilic due to the oxygen functional groups on the CB particles, enabling the following asymmetrical iron hydrogel coating and the successful construction of the LEH (Fig. 2k). It should be noted we also proposed an automatic control system for preparing the LEH with improved efficiency, where the leaf cleaning, bleaching, and conductive treatments can be managed within a chemical reactor by sequentially changing different solutions or through a continuous reactor with modular processing (more details in Supplementary Fig. 12).

We would like to further highlight the advantages of using iron hydrogel and leaf for the construction of LEH. As depicted in Fig. 2l, the storage modulus of the iron hydrogel is larger than the loss modulus in the hydrated state due to the network structure, implying the typical gel-like behavior, which is capable of locking the captured water. To demonstrate this characteristic, the water movement of the iron hydrogel on the substrate is largely restricted compared with pure hydrated LiCl (solution-like behavior, Loss modulus is higher than storage modulus in Supplementary Fig. 13), which is a strong benefit of directly using the iron hydrogel as the moisture absorbent compared with other deliquescent salts, where a further polymer encapsulation of deliquescent salts is needed to confine the water diffusion (Fig. 2m). In addition, due to the presence of metal ions within the hydrogel, it does not freeze or dry out under cold and hot environments. As shown in Supplementary Fig. 14a, there is no dramatic increase in storage modulus, indicating stability where both water reduction and freezing would lead to a noticeable rise in storage modulus. To further support this claim, we calculated the tangent (*δ*), which is the ratio of loss modulus to storage modulus. There is no increase in the low-temperature region (−10 °C to ambient) and no decrease in the high-temperature region (ambient to 50 °C) suggesting that the hydrogel remains stable and functional in both cold and hot environments (Supplementary Fig. 14b). Besides, for leaf veins, it is also beneficial in maintaining the asymmetrical water gradient across LEH. The height of the leaf veins is much higher than the surrounding mesophyll, as evidenced by height data from profilometers (Supplementary Fig. 15). Therefore, if the water were leaking from the hydrogel, the leaf veins would act as a physical “wall” and “barrier” to block water movement across the leaf. Furthermore, the leaf veins also demonstrate porous structures, which could trap the diffused water inside these pores when it contacts the leaf vein (Supplementary Fig. 16). As shown in Fig. 2o, even if the sample is placed in the ambient condition for a day and the iron hydrogel is fully hydrated, the optical and SEM images still show a clear boundary between the iron hydrogel and the untreated areas with strong contrast in the iron elemental distribution. However, a sluggish water movement is observed by the iron element mapping on the LEH without the leaf vein (Supplementary Fig. 17). Based on these advantages, the uneven distribution of hygroscopic gels led to a distinct water gradient across the LEH (Fig. 2n), which is of great importance for power generation.

### Long-term voltage output and mechanism of LEHs

**Fig. 3:**
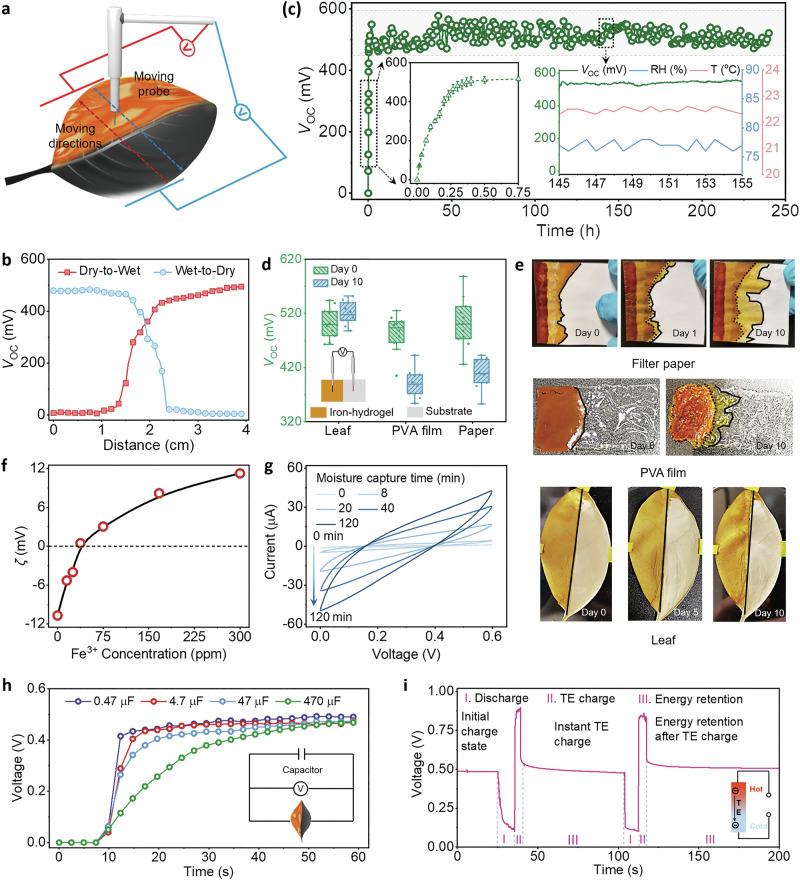
Continuous voltage output and detailed working principle of LEH. **a** Schematic illustration of the voltage measurement; one electrode is moving from the wet end to the dry end and back, while the other electrode remains stationary. **b** *V*_oc_ change of LEH during measurement, implying a voltage difference could only be generated between the wet and the dry end. **c** Continuous *V*_oc_ recording of LEH for 240 h. Insets: *V*_oc_ change of LEH during the initial moisture absorption process (left), and *V*_oc_, temperature, and relative humidity (RH) recording within 145–155 h (right). **d** The voltage output of energy harvester devices with different substrates (Device number: 10). Both paper and PVA film without the central leaf vein exhibit a voltage reduction after 10 days due to water movement, highlighting the importance of using the leaf as the substrate. **e** Digital photographs of water movement across the substrates. **f** Zeta potential (*ζ*) of CB as a function of iron concentration. **g** Operando CV measurements of LEH during the moisture absorption process. Scan rate: 0.05 V/s. **h** Discharge of LEH by capacitors with different capacitances. Electricity harvested by LEH could also be stored inside the capacitors. **i** Charging LEH through a thermoelectric panel (TE). Energy received by the TE could be maintained inside LEH, implying an application potential of LEH as a low-grade heat harvesting and storage device.

After preparation, the electrical properties of LEH were investigated. A potential difference of ~0.5 V (open-circuit voltage, *V*_oc_) was observed between the wet and dry ends across the LEH by mounting one electrode in the dry area and moving another electrode in the wet area (Fig. 3a), while the signal was quite weak when both electrodes were in the same area or the iron hydrogel was in the dehydrated state (Fig. 3b and Supplementary Fig. 18). These observations confirmed that the water gradient is the driving force for voltage output, and other interferences were ruled out by control experiments (Supplementary Fig. 19). Therefore, based on the advantages of using iron hydrogel and leaf veins mentioned above, as long as the iron hydrogel was in the hydrated state, the LEH could deliver a continuous voltage output in the range of 0.45–0.6 V for over 240 h in an open environment (Fig. 3c and Supplementary Fig. 20). While the voltage reductions on PVA and paper substrates were monitored after 10 days since the water is gradually moved to the dry sites without the hindrance of the central leaf veins (Fig. 3d, e and Supplementary Fig. 21). Moreover, the LEH performs excellent cyclic stability with nearly no performance reduction and morphology change after repeated water sorption and desorption processes for over 120 cycles (Supplementary Figs. 22, 23). More importantly, the LEH could still maintain its voltage output under ambient conditions after 9 months (Supplementary Fig. 24). These results highlighted the indispensability and advantage of using leaves for power generation.

The in-depth energy conversion routine is shown in Fig. 1b. It should be noted that the electrical behavior of these LEHs is significantly different from many other reported water-based energy generators, as stable power output was maintained under saturated moisture uptake rather than only transient power output when water droplet is in contact with the device^55,56^. We attributed its working mechanism to the hygro-contact-ionic interaction between the CB surface and iron hydrogel. Our interpretation starts with a consensus that the CB nanoparticle could lower its surface potential by building up an EDL at the solid-water interfaces when it contacts liquid water^57,58^. Such an illustration is further strengthened by voltage measurement that the dry end of the leaf served as the anode and the wet end as the cathode (Supplementary Fig. 25), and surface potential reduction between dehydrated and slightly moisturized LEH surfaces measured by Kelvin probe force microscopy (KPFM) (Supplementary Fig. 26).

To further validate cation adsorption and EDL formation, zeta potential (*ζ*) measurements and ozone treatment are carried out. It is shown in Fig. 3e that the original CB endows a negatively charged surface due to a large amount of oxygen functional groups, and an inversion of ζ is then observed with increasing iron concentration, implying the capability of cation adsorption by the CB surface. Besides, an increasing trend in voltage output with a much higher surface charge density by ozone treatment is observed, implying the formation of EDL (Supplementary Fig. 27). Based on the above results, the working mechanism is summarized: (i) Phase changes from moisture to liquid water initiated ion dissociation of the iron hydrogel; (ii) The negatively charged CB surface was induced together with EDL formation and energy storage through cations adsorption; (iii) Under external circuits, the harvested energy inside EDL is released.

Such capacitance behavior is further confirmed by operando cyclic voltammetry (CV) measurements (Fig. 3g). The measurement on a dehydrated LEH exhibits only a straight line without any capacity. With increasing moisture absorption time, an apparent curve expansion could be observed and keep constant areas when the LEH was fully hydrated (Supplementary Fig. 28). The CV curves remain in a quasi-rectangle shape and show no significant distortion under different scan rates indicating that ions adsorption process is dominated by the electric double-layer mechanism (Supplementary Fig. 29). This also enabled the LEH to work as an energy storage device, in which the EDL could be charged or discharged by external energy sources and appliances for energy storage and release. Specifically, as illustrated in Fig. 3h, the LEH could be discharged to capacitors with different capacitances with a voltage drop after discharge (Fig. 3i, Stage I). The discharged LEH could also be recharged by external energy resources for energy storage. A discharged LEH was quickly connected to a thermoelectric (TE) panel, and its voltage was swiftly increased (Stage II). Recharged energy could be successfully stored inside LEH, and a stable *V*_oc_ of 0.5 V was maintained after disconnection (Stage III). Considering TE was used to harvest low-grade wasted heat, LEH could also be repurposed for waste heat storage (Supplementary Fig. 30).

### The advantages of using the leaf and high-performance power output of LEH

**Fig. 4:**
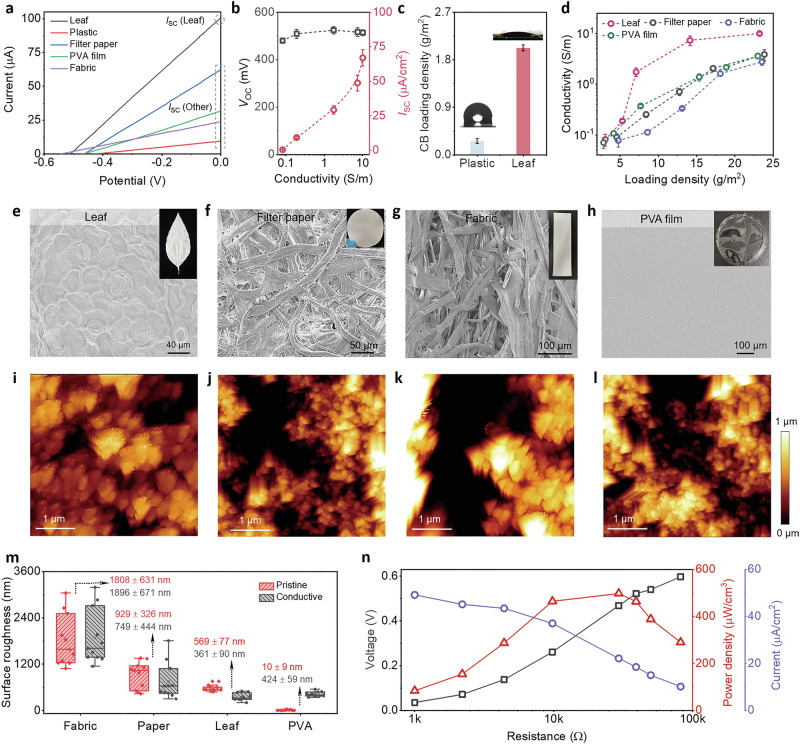
The advantage of using the leaf and high-performance power output of LEH. **a** I-V curves of energy harvester devices with different substrates. The LEH delivered the highest *I*_sc_ among others. **b** *V*_oc_ (black line) and *I*_sc_ (red line) as a function of the substrate’s electrical conductivity (Error bars: Three-time measurements). **c** CB loading content on LEH and plastic under the same dip-coating cycles. The low CB loading content on the plastic is due to its hydrophobic nature. **d** Electrical conductivity of the substrates under different CB loadings (Device number: 9). LEH endowed the highest electrical conductivity under the same CB loading content among other substrates. The electrical conductivity of PVA films could reach the same level as that of LEH only under very low CB loading content. **e**–**h** SEM images of **e** the white leaf, **f** the filter paper, **g** the fabric, and **h** the PVA film. A rough and rugged surface could be viewed on the surface of the fabric and filter paper, while the PVA film had a very flat surface. The white leaf exhibited its specific cell structures with small grooves. Inset: photos of different substrates. **i**–**l** AFM images of **i** the white leaf, **j** the filter paper, **k** the fabric, and **l** the PVA film with roughly the same CB loading density. **m** Surface roughness of the white and conductive substrates calculated through profilometer measurements. (Device Number: 11, Note that the CB loading densities of different substrates were roughly the same at around 15 g/m^2^). **n** Measured *V*_oc_, *I*_sc,_ and power output of LEH under different external resistances.

After gaining an understanding of the working principles of LEH, its electrical performance was then measured. As depicted in Fig. 4a and Supplementary Fig. 31, a pure conductive leaf exhibited ohmic behavior, while a single LEH device showed distinct power generation behavior with a *V*_oc_ of 0.5 V and *I*_sc_ of over 100 μA. The *I*_sc_ value is much higher than that of many reported self-powered energy harvesting systems, which could be a promising solution for tackling the long-lasting bottlenecks in the low power output of self-powered systems. By exchanging the leaf substrate for other commonly used substrates and repeating the same fabrication process as for LEHs, the *I*_sc_ value was found to drastically decrease to only 10–60 μA. This result implied that the substrate structures play an important role in power output.

Based on the power output mechanism, we assumed the substrate conductivity is of great importance for *I*_sc_ since more charge carriers would be involved on the substrate with high conductivity, which was further proved by a trend of *I*_sc_ improvement with conductivity (Fig. 4b) A slight reduction in *V*_oc_ within the low conductivity region was ascribed to the poor electrical network across the leaf surface. As electrical conductivity played an important role in *I*_sc_ improvement and the conductive treatment was only accomplished by dip coating of CB on the white leaf, it is anticipated that the leaf itself could endow much higher electrical conductivity under the same CB loading content compared with other substrates, as shown in Fig. 4d. This variable behavior could be ascribed to (1) the hydrophilicity of leaf is beneficial for CB particle loading, resulting in high *I*_sc_ value (Fig. 4c). (2) The specific leaf cell groove structures provided an ideal platform for CB coating and avoided CB aggregations due to small grooves on the leaf (Fig. 4e). For fabric and paper substrates, their rough and rugged surfaces may cause CB aggregation and agglomeration, which is detrimental for conductive network formation (Fig. 4f, g). For PVA film, the too flat and smooth surface is also harmful to the conductive network formation (Fig. 4h and Supplementary Fig. 32), which is consistent with the results in Fig. 4d.

These interpretations were further consolidated by tapping these substrates after CB coating by atomic force microscopy (AFM, Fig. 4i–l). A well-distributed CB network was clearly viewed on LEH, while CB aggregation was observed on the other substrates, which was further confirmed by the quantitative measurement of the surface roughness. The white leaf had moderate surface roughness. The roughness of the leaf was apparently reduced after CB coating, implying CB particles were well-distributed on the leaf surface. However, other substrates endowed either too high or too low surface roughness (Fig. 4m and Supplementary Fig. 33). (3) In addition, after CB coating, a much lower thickness for the LEH (162 μm, fabric 327 μm) was another advantage since an increased volumetric power density could be achieved by stacking more devices in parallel (Supplementary Fig. 34). Based on these advantages of using the leaf as the substrate, the LEH delivered a remarkable optimal power density of 497 μW/cm^3^ (areal: 12.43 μW/cm^2^) by connecting to external resistors, outperforming the majority of reported self-powered energy harvester (Fig. 4n and Supplementary Tables 1, 2). Some other influential factors on LEH were highlighted in Supplementary Figs. 35–38 and Supplementary Note 4.

It should be highlighted that the proposed fabrication routine could be applied to different types of fallen leaves, including Pubescent Holly, Bodhi, Maple, and Lilac leaves. After standard leaf bleaching treatment, the specific leaf cell structures with small grooves are all well observed together (Supplementary Fig. 39a) with moderate surface roughness for carbon black accommodation (Supplementary Fig. 39b), which is the key success of our approach. Therefore, no specific performance variation is found within different kinds of leaves (Supplementary Fig. 39c). In addition, the developed LEH exhibits a wide operational range. As depicted in Supplementary Fig. 40, our LEH can quickly build up voltage output even at medium relative humidity (40% RH) due to its porous network, which enables fast moisture sorption kinetics and capacity. This performance surpasses that of the majority of metal and coordination-based hygroscopic materials (see Supplementary Fig. 40 and Supplementary Table 3 for more details).

### Self-regeneration of LEHs for continuous current output and practical applications

**Fig. 5:**
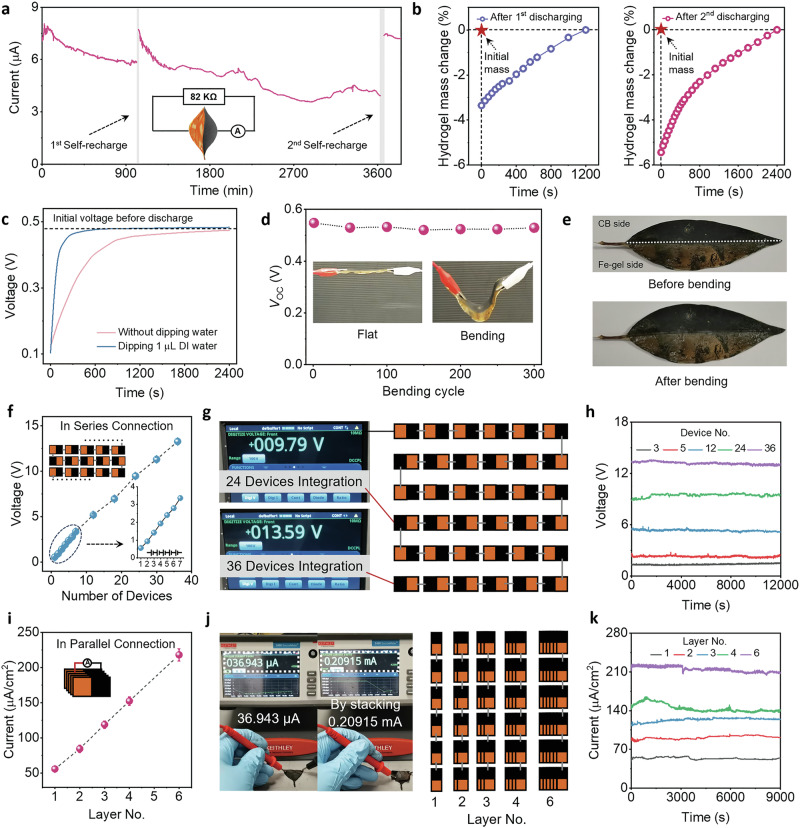
Self-regeneration and practical applications of LEH. **a** Two continuous charge-self-recharge cycles. LEH was first discharged by connecting with 82 kΩ external resistance for 16 h, where the output current was monitored, followed by 20 min self-regeneration. Then, the LEH was discharged for 40 h, followed by 40 min self-regeneration, implying the practical regenerative power output of LEH. The duration of the self-regeneration was positively correlated with the discharging time. **b** Mass change during the two times of self-regeneration of LEH. A much bigger mass change with longer self-regeneration time was observed after LEH discharge over a longer time. **c** Voltage of LEH during self-regeneration. By dipping 1 μL water droplets on LEH after discharge, the duration of self-regeneration was largely decreased. **d** Cyclic bending test of LEH. No apparent performance reduction was shown during the test. **e** Digital photographs of LEH before and after 300 bending cycles. Scalability of LEHs by parallel and series connections with improved **f** *V*_oc_ and **i** *I*_sc_ output. Series connections could be realized by connecting the cathode and the anode of LEH alternatively. **g**,** h** The power panel is developed through the integration of 36 devices on a flexible substrate with ~13 V voltage output. **j** The parallel connection could be performed by stacking multiple LEHs. **k** Current output could reach the magnitude of a milliampere (mA) by stacking multiple LEHs.

After LEH discharge, aside from recharging by external energy resources, it was observed that the device could spontaneously and repeatedly recover its energy by simply putting it in the ambient environment, similar to protein-based self-powered energy harvesters (Supplementary Fig. 41)^24,59^. As depicted by Fig. 5a, by connecting LEH with a load resistor, more than 40 h of continuous current output was observed, and the device was then quickly restored to its original status within 40 min by removing the load resistor and leaving it in the ambient condition. Besides, the duration of the self-regeneration was positively correlated with the discharging time. Further experimental observations suggested that the self-regeneration behavior originated from the dynamic water exchange between the ambient environment and the LEH. While a small mass change, it played a critical role in driving the self-regeneration process (Fig. 5b). Furthermore, the magnitude of mass change was also related to the discharging time. Therefore, a sluggish dehydration process took place during LEH discharge, and the desorbed water was captured again during self-regeneration, which was further corroborated by a tiny temperature difference between LEH discharge and self-regeneration (Supplementary Fig. 42). Besides, the duration of the self-regeneration process was largely decreased by dipping 1 μL water droplets on LEH after discharge, thus further validating the dynamic water exchange process during LEH discharge and self-regeneration (Fig. 5c). Taking advantage of self-regeneration, an LEH could maintain a continuous current output, and therefore could be employed for electricity accumulation by continuously harvesting energy from ambient air (Supplementary Fig. 43).

Strong mechanical strength is a prerequisite for the practical application. Compared with the brittleness of fallen leaves, no apparent cracks or performance degradation of LEH was identified after 300 cycles of bending, which could be ascribed to the addition of plasticizer and carbon black as fillers during leaf treatment to improve mechanical strength (Fig. 5d, Supplementary Fig. 44, and Supplementary Video 1). Stress-strain curves of LEH, pristine fallen leaves, and other fiber-based materials were further measured. As shown in Supplementary Fig. 45, LEH demonstrated satisfactory mechanical strength, modulus, and elongation at break compared with other fiber-based materials. The LEH also shows better mechanical properties than pristine leaves. Together with weight-lifting experiments that 0.4 g LEH could lift 100 g weight, we believe LEH endows satisfactory mechanical strength for diverse application scenarios (Supplementary Fig. 46). A cost analysis indicates developing 1 kg of LEH requires $4.88, and such value could be further reduced with further optimization of the preparation process. (Supplementary Note 5 for more analysis). Moreover, LEHs exhibited superior scalability on power generation due to readily series connection, and the *V*_oc_ could be readily improved to over 3 V with 7 LEHs (Fig. 5f and Supplementary Fig. 47). Together with high current density output of LEH (~50 μA/cm^2^), the power output could meet the usage requirement of small electronics, including light emitting diodes, calculators and digital clocks (Supplementary Figs. 48–50 and Supplementary Videos 2–4). It should be noted that continuous power output could be delivered by LEHs due to their self-regeneration effects, thus, all these appliances could be powered for long-term use^60^. In addition, the voltage output could be further scaled up by integrating several dozens of LEH on a flexible substrate in a small area, and a continuous *V*_oc_ signal with over 13 V was observed (Fig. 5g, h and Supplementary Video 5) and no performance reduction was detected under bending and twisting conditions (Supplementary Fig. 51 and Supplementary Video 6). The current output of the LEHs could also be increased by simply increasing the area of the iron hydrogel on the LEH (Supplementary Fig. 52) or in-parallel connections through stacking multiple LEH devices to reach milliampere (mA) magnitudes (0.2 mA, Fig. 5i–k).

### Environmental benefits of LEHs

To evaluate the environmental impact of our approach, we conducted a cradle-to-gate life-cycle assessment (LCA) to quantify the environmental impacts of LEH and compared the results to commonly used carbon-based energy harvesters (Supplementary Note 6). As depicted in Supplementary Fig. 53, aside from ecotoxicity, the environmental impacts of LEHs were at least one order of magnitude lower than carbon-based energy harvesters. The usage of acetic acid during LEH production was the major contributor (~70%) to ecotoxicity and led to a 3.3-fold increase, however, the value was still slightly lower than that of carbon-based energy harvesters. A contribution analysis was then carried out considering other aspects of environmental impacts, and the results indicated that the usage of electricity for leaf bleaching and conductive treatment is the main contributor to environmental categories such as global warming potential, acidification, eutrophication and particular matters (~70%). The human toxicity was mostly driven by the usage of ethanol and ethanolamine. As the electricity in this analysis was assumed to originate from coal-fired power, it is anticipated that the environmental impacts could be further reduced with the use of clean energy resources, which in turn would maximize the environmental benefits of LEH. For the rest of the environmental impacts, the usage of sodium chlorite during leaf bleaching contributed to global warming and eutrophication, while the introduction of CB during conductive treatment raised the environmental impacts of acidification and particular matters.

## Discussion

In summary, inspired by the specific structures of leaves, we have proposed a facile approach to convert fallen leaves into green energy harvesters. The LEHs could be readily prepared by leaf bleaching, conductive treatment, and asymmetrical hygroscopic iron hydrogel coating on one end of the leaf through facile fabrication processes. After moisture capture, the developed iron hydrogel was capable of locking water inside the hydrogel due to the network structure, and the water movement was further cut off by leaf veins. Therefore, a distinguished water gradient was established across the LEH, followed by ion dislocations, liquid water contact, and EDL formation, showing capacitance-like behavior for energy charging and discharging. The specific leaf cell structure with small grooves enabled a uniform carbon coating instead of aggregations, thus delivering a high current density *I*_sc_ (~50 μA/cm^2^) and power output (12.43 μW/cm^2^ and 497 μW/cm^3^) under external resistance, outperforming the majority of self-powered energy harvesting devices with different substrates. These are key advances in using leaves as substrates for energy harvesting. A self-regeneration phenomenon was achieved, which depends on dynamic water absorption and desorption, guaranteeing the continuous current output.

Before closing, we acknowledge that the leaf-based energy harvester at the current stage has several limitations regarding device preparation, integration, and post-treatment after the device’s lifetime. For device preparation, the current approach still requires a two-step chemical treatment and precisely asymmetric coating of hygroscopic hydrogel on one side of fallen leaves. The chemical uses increase the environmental impact and multiple steps with complicated processes require more labor and energy costs, and with potentially lower economic and environmental benefits. Therefore, future orientations could focus on further optimizing the preparation routine with a more straightforward and facile approach to the valorization of fallen leaves with low environmental impact.

For device preparation, limited to the small size of a piece of fallen leaves (roughly less than 50 cm^2^), the power output still has an upper limit. The large-scale fabrication requires a complicated parallel and series connection. For instance, making 1 m^2^ of energy harvesters will require the separate processing of 200 leaves. Although an automatic assembly line approach is proposed to increase the efficiency of large-scale fabrication, the hydrogel viscosity and the parameters of this machine still require further investigation and optimization. Our original aim for this work is to increase the power output in the unit area (power density) so that the harvested energy is high enough to serve as a power source for wearable sensors and patches, since the miniature and continuous power output at several microwatts is always required.

After the device's service lifetime, whether the leaf-based energy harvester could be decomposed remains a question. One recent work utilized living leaves directly as energy harvests, which could be a promising solution and future direction. However, the corresponding low power output and the reliance on water transpiration still need further optimization^61^. Developing a bioresorbable energy harvester could be a promising solution for future orientation. In addition, the life-cycle assessment and the cost analysis are simplified and less than the real application scenarios. For environmental impacts, our analysis only includes the use of raw materials during the fabrication process without consideration of the environmental impact generated by the required energy and labor force. Besides, our comparison is carried out with only carbon-based energy harvesters, further comparison is needed to carefully evaluate the overall sustainability. Regarding the cost analysis of large-scale fabrication, materials waste, equipment usage, and laboring are not included in the cost, and the realistic cost will be higher than our estimation. Further reduction in environmental impacts and cost of leaf-based energy harvesters could stand out the overall sustainability and practicability of this approach.

## Methods

### Preparation of iron hydrogel

Iron chloride hexahydrate (FeCl_3_·6H_2_O), ethanolamine (EA), and ethanol were all purchased from Sigma Aldrich and used without further purification. 2.16 g FeCl_3_·6H_2_O was first dissolved in 10 ml ethanol under ultrasonication for 20 min. Later, 240 μL EA was added to the solution and ultrasonicated for a further 20 min to obtain the iron hydrogel.

### Preparation of LEH

Firstly, fallen leaves were collected and disinfected before usage. Then, 30 g leaves were treated with 500 ml 0.6 wt% acidified sodium chlorite solution at 75 °C for 1 h. The treatment was repeated five times and the leaves were washed with distilled water after the bleaching treatment. Later, 10 g triethyl citrate was dispersed in 200 ml water, and the leaves were placed into this solution for 12 h. The white leaves were finally obtained by pressing with glass sheets and drying under indoor environmental conditions.

CB ink was prepared by dispersing 0.4 g CB (Alfa Aesar) and 0.6 g sodium dodecyl benzene sulfonate (SDBS, Sigma Aldrich) into the water with 3 h of ultrasonication to obtain uniform ink. Conductive treatment was then carried out by repeatedly dip-coating CB ink onto the white leaf and drying at 70 °C until a uniform distribution of CB on the leaf surface was achieved (usually at around 15 g/m^2^). As prepared iron hydrogel was then dropped on the conductive leaf with moderate volume, depending on the size of the leaf, and dried at 70 ^o^C to finish the fabrication of the LEH. The LEH was then left in ambient conditions for moisture absorption (RH: ~75%, 25 °C).

The power panel in Fig. 5g, j was developed by cutting a conductive leaf into several pieces but kept the central vein on each piece and followed the same protocol to fabricate LEH. Then, the prepared LEHs were glued on a flexible polyethylene terephthalate (PET) substrate with the aid of stainless foil to connect each LEH.

### Characterization and measurement of LEH

The humidity is well controlled during the experiment. Our lab is under nearly constant relative humidity (~74–78% RH) as calibrated by several Kestrel 500 Environmental Meters, which is also the average relative humidity in Singapore. To avoid the fluctuation, batches of devices were fabricated, and many repeated measurements were conducted with many samples from different batches. That is the reason many error bars are shown in our data. For the measurements that require precise humidity control (e.g., the device performance as a function of relative humidity and different temperatures, water uptake of hygroscopic hydrogel), it is conducted within a humidity chamber (KMF 115, Binder) with calibration by a Kestrel 5000 Environmental Meter. A hole on the side of the humidity chamber could guarantee the connection between outside equipment (e.g., multimeter, balance) and inside samples. For those samples measured in the lab environment, we will also remeasure them in the humidity chamber to avoid the discrepancy in our data. The measurements were conducted at relative humidity within 74–78% RH, unless with extra humidity data and illustrations in specific figures.

FT-IR was measured by an Agilent Cary 660 spectrometer. All solutions were dropped on potassium bromide (KBr) pellets and dried before measurements. XPS was conducted using a Kratos Axis Ultra DLD spectrometer. The microstructure of the iron hydrogel and leaves were characterized by SEM (Zeiss Supra 40VP) with EDS mapping. Water uptake measurements via net mass change of the iron hydrogel, FeCl_3_.6H_2_O, and EA were all conducted using an electronic balance (Sartorius CP224S). Water distribution across LEH was performed in a similar way by calculating the initial and final mass after moisture absorption. Specifically, LEH was cut into several pieces under dehydrated conditions, and the mass of the individual pieces was measured. After moisture absorption, the mass change of each small piece was recorded. The water absorption ability of the iron hydrogel under different RHs, namely, the water absorption isotherm, was measured by a dynamic vapor sorption analyzer (Aquadyne DVS) at 25–55 °C. The porosity of the green and white leaves was determined by X-ray 3D microscopy (Nanovoxel-2100, Sanying Precision Instruments Co., Ltd., China). The surface roughness of the leaf and fabric was calculated using data from a profilometer (Alpha-Step IQ). Linear sweep voltammetry (LSV), cyclic voltammetry (CV, cut-off voltage: 0–0.6 V), and Electrochemical impedance spectroscopy (EIS) measurements were obtained via an electrochemical workstation (CHI660E, CH Instruments, Inc.). The CV and LSV are carried out under ambient conditions at relative humidity within 74–78% RH. KPFM measurements were performed using a Dimension Icon (Bruker Nano Surfaces). The Zeta potential of CB particles was measured with a NanoBrook ZetaPALS (Brookhaven). A dual-range analytical balance was employed to monitor the small mass change of LEH during self-regeneration (Shimadzu AP125WD). Frequency sweep tests were measured on an automatic rheometer (MCR302, Anton Paar). TGA was carried out on a TGA Q500 (TA Instruments). The moisture absorption processes of the iron hydrogel in ambient conditions were monitored with an optical microscope (Nikon Eclipse LV1000 and Digital Sight DS-U1). Temperature and humidity were monitored using a Kestrel 5000 Environmental Meter. The small temperature change during self-recharge was monitored by a UT 322 thermometer (UNI-T). Water contact angle measurements were carried out on a Drop Shape Analyzer (DSA 30, Krüss). Ozone treatment of LEH was carried out by a digital UV ozone system (Novascan). AFM measurement was carried out by JPK instruments with the model of Nano Wizard 3. The power density of LEH was measured by connecting it with different resistors. By measuring the voltage between two ends of the resistors, the corresponding current density and areal power density could be calculated by Ohm's and Joule’s law. The volumetric density is calculated by multiple areal power density with 40, where 40 LEHs could be integrated within a 1 cm height in practical applications (Supplementary Fig. 34b).

### Reporting summary

Further information on research design is available in the Nature Portfolio Reporting Summary linked to this article.

## Supplementary information

Supplementary Information

Description of Additional Supplementary Files

Supplementary Video 1

Supplementary Video 2

Supplementary Video 3

Supplementary Video 4

Supplementary Video 5

Supplementary Video 6

Reporting Summary

Transparent Peer Review file

## Source data

Source Data

## Data Availability

The data that supports the findings of the study are included in the main text and supplementary information files. Source data are provided with this paper.
