# Supplementary material for: Leaf-based energy harvesting and storage utilizing hygroscopic iron hydrogel for continuous power generation
Source: Nat Commun. 2025 Jun 6;16:5267. doi: 10.1038/s41467-025-60341-z (PMC12144242; doi:10.1038/s41467-025-60341-z)
Supplement: Supplementary file 1 — Supplementary Information [file 41467_2025_60341_MOESM1_ESM.pdf]

Supplementary Information

Leaf-Based Energy Harvesting and Storage Utilizing Hygroscopic Iron Hydrogel for Continuous Power Generation

Shuai Guo[1,#], Yaoxin Zhang[6,#], Zhen Yu[1], Ming Dai[3], Xuanchen Liu[3], Hongbo Wang[2], Siqi Liu[1], J. Justin Koh[10], Wanxin Sun[9], Yuanping Feng[8], Yuanzheng Chen[7], Lin Yang[11], Peng Sun[12], Geyu Lu[12], Cunjiang Yu[5,13,14,15], Wenshuai Chen[3,*], Stefaan De Wolf[4,*], Zuankai Wang[2,*], and Swee Ching Tan[1,*]

[1]Department of Materials Science and Engineering, 9 Engineering Drive 1, Singapore 117575, Singapore; [2]Department of Mechanical Engineering, The Hong Kong Polytechnic University, Hong Kong, P.R. China; [3]Key Laboratory of Bio-based Material Science and Technology, Ministry of Education, Northeast Forestry University, Harbin 150040, P.R. China; [4]KAUST Solar Center (KSC), Physical and Engineering Division (PSE), King Abdullah University of Science and Technology (KAUST), Thuwal 23955-6900, Kingdom of Saudi Arabia; [5]Department of Electrical and Computer Engineering, University of Illinois Urbana-Champaign, Urbana, IL 61801, USA; [6]China-UK Low Carbon College, Shanghai Jiao Tong University, Shanghai 201306, P.R. China; [7]School of Physical Science and Technology, Key Laboratory of Advanced Technologies of Materials, Southwest Jiaotong University, Chengdu 610031, P.R. China; [8]Department of Physics, National University of Singapore, 2 Science Drive 3, Singapore 117551, Singapore; [9]Division of Nano Surfaces, Bruker Corporation, 11 Biopolis Way, Singapore 138667, Singapore; [10]Institute of Materials Research and Engineering (IMRE), Agency for Science, Technology and Research (A*STAR) , 2 Fusionopolis Way, Innovis #08-03, Singapore 138634, Republic of Singapore; [11]Key Laboratory of Optoelectronic Technology and System of Ministry of Education, College of Optoelectronic Engineering, Chongqing University, Chongqing, 400044, P. R. China; [12]State Key Laboratory on Integrated Optoelectronics, College of Electronic Science and Engineering, Jilin University, Changchun,

China; [13]Department of Materials Science and Engineering, University of Illinois, Urbana-Champaign, Urbana, IL, USA. [14]Department of Mechanical Science and Engineering, University of Illinois, Urbana-Champaign, Urbana, IL, USA. [15]Department of Bioengineering, Materials Research Laboratory, Beckman Institute for Advanced Science and Technology, Nick Holonyak Micro and Nanotechnology Laboratory, University of Illinois, Urbana-Champaign, Urbana, IL, USA. [#]These authors contributed equally: Shuai Guo, Yaoxin Zhang.

*Corresponding authors: Wenshuai Chen (chenwenshuai@nefu.edu.cn), Stefaan De Wolf (stefaan.dewolf@kaust.edu.sa), Zuankai Wang (zk.wang@polyu.edu.hk), Swee Ching Tan (msetansc@nus.edu.sg)

Contents

1. Supplementary Note 1-6. 4
2. Supplementary Fig. 1-53 14
3. Supplementary Table 1-3. 67
4. Supplementary References 70

Supplementary Note 1-6

Supplementary Note 1. Evaluation standard of the comparison between LEH and other self-powered energy harvester devices in Supplementary Fig. 1

The evaluation of the power output is based on areal power density. For LEH, the value reached 12.43 μW/cm^2 with continuous voltage output. While metal and bio-polymer-based energy harvesting devices may achieve the same or higher voltage levels, their current output density is often limited to less than 10 μA/cm^2, resulting in significantly lower overall power output [1-3]. Two representative examples include:

- Protein-based nanowires, which yielded a power density of 4.8 μW/cm^2.[2]
- Bilayer biopolymer film (Poly(diallyldimethylammonium chloride) (PDDA), Polystyrene sulfonic acid (PSSA)) with a power density of 2.5 μW/cm^2. [4]

Regarding carbon-based energy harvesters, while some devices could achieve current output densities over 0.1 mA/cm^2 and power outputs higher than LEH, their voltage output is typically limited to less than 200 mV. Importantly, these devices usually generate transient or intermittent power output[4], requiring a much longer time to regenerate the power output. Although carbon-based energy harvesters receive a high score in certain metrics, their intermittent power output raises concerns regarding practical applications.

The evaluation of the cost is based on the data from Alibaba.com. The total cost of our leaf-based energy harvester consists of raw materials fallen leaves (if purchased: US$3.6/kg), fabrication and processing cost (US$1.28), and device integration cost (US$2.12). It is important to note that the device integration cost applies to all types of energy harvesters for scalable production. However, the cost of metal nanoparticles for energy harvesters from moisture (e.g., titanium oxide, TiO_2) is at least US$45/kg [1], while the carbon-based energy harvesters (e.g., graphene) is at least US$10/kg [5]. Additionally, the costs for PDDA and PSSA polymer solutions are significantly higher, starting from US$190/kg and US$490/kg, respectively. These costs are much higher than the cost of fallen leaves. It should be mentioned that the cost of fallen leaves could be further reduced through collection. The cost used here (also including company profit) is the cost from Tongbai County Huachuang Agricultural

Development Co., LTD, a company that specializes in clean fallen leaves collection, storage, washing, and transportation.

The environmental impacts of LEH compared to carbon-based energy harvesters are evaluated in Supplementary Fig. 53 and Supplementary Note 6. The environmental impacts of LEH are at least one order of magnitude lower than that of other energy harvesters except in terms of ecotoxicity. The ecotoxicity of our leaf-based energy harvesters remains lower than that of other types of energy harvesters. The sustainability of raw materials is enhanced by the processing and recyclability of fallen leaves which are biodegradable and do not require any pre-processing (e.g., graphene requires the Hummers method to convert into graphene oxide, and TiO_2 needs sieving). Scaleable production and eco-friendly fabrication processes are key to evaluating the sustainability and cost of the fabrication process. For LEH, only commonly used methods such as dip coating, ultrasonic processing, and oven drying are needed, and the raw materials are the most economical compared to other types of materials. Other types of energy harvesters, usually require costly device fabrication processes, such as high-power laser engraving[6-8], which increase the complexity and reduce device scaleability. To further demonstrate the scalability of our techniques, we propose an automatic assembly line with a fixed printer to pinpoint coating hydrogel on leaves, enabling fully automatic device fabrication (Supplementary Fig. 12).

Finally, while we recognize some subjective factors that exist in comparing LEH with other types of energy harvesters based on key sustainability and device performance metrics, our aim in Supplementary Fig. 1 is to highlight the low cost and the continuous power output of LEH.

Supplementary Note 2. Theoretical simulations

The density functional theory (DFT) simulations of $FeCl_3 \cdot 6H_2O$, iron hydrogel, and their molecular absorptions were performed using the Vienna ab initio simulation package (VASP)[9]. In the framework of DFT, the structural optimizations were achieved using an exchange-correlation functional treated with generalized gradient approximation (GGA) using the Perdew-Burke-Ernzerhof density functional[10]. The electron projector-augmented wave (PAW) method was employed with PAW potentials[11]. An energy cutoff of 750 eV for the plane-wave expansion was adopted and appropriate Monkhorst-Pack k meshes of uniform spacing of $2\pi\times0.03$ $Å^{-1}$ were chosen during ab initio electronic-structure calculations[12]. The van der Waals density functional was adopted to treat the weak interactions in the structure. The water molecule embedding energy (ΔE) of the iron hydrogel was calculated by the definition: ΔE = E(iron hydrogel + H_2O) – E(iron hydrogel) – E(H_2O). E(iron hydrogel + H_2O) and E(iron hydrogel) are the total energies of the iron hydrogel system embedded with and without one water molecule, respectively. All structural relaxations were converged to within 0.01 eV/Å for each atom, and total energies were converged to within 0.1×10^{-5} eV.

Supplementary Note 3. Fourier-transform infrared spectroscopy (FT-IR), X-ray photoelectron spectroscopy (XPS), and scanning electron microscopy (SEM) analyses of the structure of iron hydrogel

As shown in Supplementary Fig. 2, the FT-IR spectrum of the iron hydrogel shows two distinct bands at 1058 cm^{-1} (ν(C-N)) and 995 cm^{-1} (ν(C-O)) which are redshifted compared with ethanolamine (EA) (1070 cm^{-1} (ν(C-N)) 1025 cm^{-1}(ν(C-O))[13]. Upon coordination, a blue shift in the FT-IR spectrum of the iron hydrogel was observed for the ν(N-H) (1614 cm^{-1}) and ν(O-H) (1379 cm^{-1}) bands in comparison to those of EA (1608 cm^{-1} and 1374 cm^{-1}, respectively)[14]. These results indicated that iron atoms were coordinated with oxygen and nitrogen on EA, which was in agreement with our simulation results. XPS was also used to reveal the composition of the iron hydrogel; five primary elements of $FeCl_3 \cdot 6H_2O$ and EA (C, N, O, Fe, Cl) were observed in the survey spectrum. The high-resolution Fe 2*p* spectrum showed two peaks at 724 and 711 eV, corresponding to Fe $2p_{1/2}$ and Fe $2p_{3/2}$, respectively[15]. Moreover, splitting of the N 1*s* spectrum at 400 and 398 eV was seen, indicating the strong coordination between Fe and EA together with charge transfer between the nitrogen of EA and $FeCl_3 \cdot 6H_2O$ (Supplementary Fig. 3)[16]. SEM was applied to reveal the microstructure of iron hydrogel. Due to the strong coordination interactions between EA and the iron precursor, the iron hydrogel showed a distinct entangled network structure as illustrated in Supplementary Fig. 4, which aided moisture capture and release, resulting in fast absorption and desorption kinetics. The uniform distributions of Fe, C, O, and Cl on the energy-dispersive X-ray spectroscopy (EDS) spectrum could be clearly observed, however, by contrast, N was mainly located on the network skeleton, further proving the coordination interaction between EA and $FeCl_3 \cdot 6H_2O$ (Supplementary Fig. 5).

Supplementary Note 4. Factors that influence the LEH performance

According to the LEH mechanism, the V_{oc} was highly dependent on the water gradient. Therefore, the performance of LEHs inevitably relied on environmental humidity. As depicted in Supplementary Fig. 35, the LEH started to demonstrate a voltage response at ~20% RH, which was equivalent to the starting RH of moisture uptake of the iron hydrogel. The optimal voltage output could be realized within a wide humidity range from 30-95% RH, which is also the range in which the moisture uptake of the iron hydrogel began to increase dramatically. Further increases in RH will introduce contradictory effects on voltage output since the trace amount of surfactant existing in the CB coating (sodium dodecyl benzene sulfonate, SDBS) could capture moisture at such high RH, destroying the water gradient (Supplementary Fig. 36)[17]. Besides, the voltage of LEH could be quickly built up within 30 minutes at 30, 60, and 90% RH. The electrical performance of LEH under different temperatures was then investigated (Supplementary Fig. 37a). The voltage output exhibited stabilized performance from 3-35 °C, while an increasing trend of current output densities was observed, which was ascribed to the elevation of LEH capacitance with the increasing temperature (Supplementary Fig. 37b), that is caused by the improvement in both ionic and electrical conductivity (Supplementary Fig. 37c). The water loss caused by the enhanced temperature has minimal effects on LEH performance since the iron hydrogel could still maintain superior moisture uptake capacity at 35 °C. Although the current output densities are varied with different temperatures, the LEH power output could still reach a satisfactory level, implying a wide operation temperature range of LEH. In addition, an all-day outdoor test was then carried out to validate our suspicion. Regardless of the variation of RH and temperature over the period of one day, the LEH performance remained virtually unchanged (Supplementary Fig. 38).

Supplementary Note 5. Cost analysis of LEH

Items		Unit Price (USD)
Chemicals	Sodium Chlorite ($NaClO_2$)	$0.499/kg
	Ethanol (C_2H_5OH)	$1/kg
	Water	~$0.34/ton (Price from Harbin)
	SDBS	$0.8/kg
	Iron(III) chloride hexahydrate ($FeCl_3$ $6H_2O$)	$1/kg
	Acetic Acid (CH_3COOH)	$0.59/kg
	Carbon Black	$0.4/kg
	Triethyl Citrate	$1.23/kg
	Ethanolamine	$1.59/kg
Raw Materials	Fallen Leaves	~$3.6/kg (Purchase, Price from Alibaba)
Device Fabrication	Stainless Steel Tape	$10/m^2 Price from Alibaba
	Poly(ethylene terephthalate), PET	~$1.3/kg (Price from Ocean Polymer Materials co, Ltd)

The fabrication cost for 1 kg LEH was calculated as below:

**Items**		**Unit Price (USD)**	**Amount**	**Total Price (USD)**
Chemicals	Sodium Chlorite ($NaClO_2$)	\$0.499/kg	0.5 kg	\$0.2495
	Ethanol (C_2H_5OH)	\$1/kg	0.162 kg	\$0.162
	Water	~\$0.34/ton	0.1 ton	\$0.034
	SDBS	\$0.8/kg	0.042 kg	\$0.0336
	Iron(III) chloride hexahydrate ($FeCl_3$ $6H_2O$)	\$1/kg	0.027 kg	\$0.027
	Acetic Acid (CH_3COOH)	\$0.59/kg	0.095 kg	\$0.056
	Carbon Black	\$0.4/kg	0.042 kg	\$0.0168
	Triethyl Citrate	\$1.23/kg	0.33 kg	\$0.4059
	Ethanolamine	\$1.59/kg	0.003 kg	\$0.00477
Raw Materials	Fallen Leaves	~\$3.6/kg	1 kg	~3.6
Device Fabrication	Stainless Steel Tape	\$10/m^2 Price from Alibaba	0.1 m^2	~1
Device Fabrication	Poly(ethylene terephthalate), PET	~\$1.3/kg (Price from Ocean Polymer Materials co, Ltd)	0.8625 kg	~1.12
Electricity		~\$0.08 $(kW\ h)^{-1}$	3.8 kW h	\$0.304
Total		At most \$7/kg (\$3.6 for fallen leaves and \$2.12 for device integration)		

Cost analysis for fabricating 1 kg LEH. Prices are based on the bulk purchase of raw materials. The electricity is mainly consumed by the oven (0.3 kWh, 2 hours) hot plate (0.1 kWh, 5 hours), and ultrasonic bath (0.2 kWh, 40 mins) during the delignify process and iron hydrogel

fabrication. It should be noted that the cost could be further reduced through the collection of fallen leaves. Actually, in this work, the fallen leaves are collected directly instead of purchased, meaning that the actual cost could be largely reduced. However, to find out the maximum cost for our fabrication process, we used the purchase cost of fallen leaves to cover the potential cost of collecting, selecting, storing, transporting, and washing fallen leaves. We believe this cost is applied to all leaf processing strategies or research based on fallen leaves. Besides, it should be highlighted that the cost also contains the device fabrication and integration. In detail, 1 kg of fallen leaves could be fabricated into ~2300 pieces (assume 2000 could work well and the rest 300 are defective samples) of leaf-based energy harvesters (LEHs). By stacking 2 pieces of LEHs together in parallel, the total leaf-based energy harvesters could occupy ~25 m^2 (1 LEH is ~10 cm^2). The thickness and the density of poly(ethylene terephthalate) (PET) substrate are 0.05 mm and 1.38 g/m^3, respectively. Therefore, the PET requires ~4 kg. For the stainless steel tape, it is used to connect these LEHs, the stainless steel is cut into 10 mm×10 mm each and it requires 0.1 m^2 in total. Therefore, it requires ~US$2.12 for device integration. We believe this cost (~US$2.12) is applied to all self-powered systems for device integration since the flexible substrate and connection tape are indispensable. The total cost of making LEHs into large-scale and real applications is ~US$7/kg, which includes US$3.6 for 1 kg of fallen leaves, US$1.28 for leaf processing and LEH preparation, and US$2.12 for device integrations.

Before closing, we also want to acknowledge that for large-scale fabrication, materials waste, equipment usage, and laboring are not included in the cost and the realistic cost will be higher than our analysis. Further reduction in environmental impacts and cost of leaf-based energy harvesters could stand out the overall sustainability and practicability of this approach.

Supplementary Note 6. Life cycle assessment (LCA) of the leaf-based energy harvester (LEH) and carbon-based energy harvester.

The life cycle inventory (LCI) data of upstream production chemicals, electricity, and water were collected from the Ecoinvent v3.8 database[18], literature[19-21], and U.S. Environmental Protection Agency (EPA) website[22]. For the leaf-based energy harvester, the following chemicals were included: (1) Sodium chlorite, acetic acid, and triethyl citrate for leaf processing; (2) Carbon black (CB) and sodium dodecylbenzene sulfonate for CB ink preparation; (3) Iron(III) chloride hexahydrate and ethanolamine for iron hydrogel synthesis. Water and ethanol were used as solvents during LEH fabrication. As the LCI data of sodium chlorite cannot be obtained directly from the database, we replaced it with an analog, sodium hypochlorite, that could cause roughly the same environmental impacts during the production phase, which have been well-proved by previous literature on the LCA of wood production[19-21]. The LCI data of other chemicals were obtained through the database. The life cycle impact assessment (LCIA) was performed using the tool for the reduction and assessment of chemical and other environmental impacts (TRACI) developed by the U.S. Environmental Protection Agency[22]. For the carbon-based energy harvester, Hummer's process on graphene oxide was applied in the material preparation, and its environmental impact has been thoroughly analyzed by previous literature. We collected LCA data directly from the literature and compared it with the LCI data of LEH[23,24]. Aside from ecotoxicity, the environmental impacts of LEH were at least 10 times lower than those of the carbon-based energy harvester.

We also want to highlight that our analysis only includes the use of raw materials during the fabrication process without consideration of the environmental impact generated by the required energy and labor force. Besides, our comparison is carried out with only carbon-based energy harvesters, further comparison is needed to carefully evaluate the overall sustainability.

Some data collected for LCA of leaf-based energy harvester

Acetate Acid: 3.71×10^{-3} kg CO_2 eq/kg, 1.9×10^{-2} kg N eq/kg, 12.6 kg CTU_e/kg.

Carbon Black: 1.97 kg CO_2 eq/kg, 3.3×10^{-3} kg SO_2 eq/kg, 1.05×10^{-3} kg N eq/kg, 1×10^{-3} kg PM

2.5 eq/kg, 1.67×10^{-2} kg CTU_e/kg.

Ethanol: 0.736 kg CO_2 eq/kg, 0.604 kg CTU_e/kg, 3.64×10^{-9} kg CTU_h/kg.

SDBS: 9.07×10^{-3} kg CO_2 eq/kg, 2.93×10^{-4} kg SO_2 eq/kg, 1×10^{-4} kg N eq/kg, 1.64×10^{-4} kg PM 2.5 eq/kg, 4.33 kg CTU_e/kg.

$FeCl_3$: 1.04×10^{-2} kg CO_2 eq/kg, 4.51 kg CTU_e/kg.

Ethanolamine: 7.19×10^{-2} kg CO_2 eq/kg, 1.94×10^{-2} kg SO_2/kg, 4×10^{-2} kg CTU_e/kg, 2.04×10^{-9} kg CTU_h/kg.

Sodium Chlorite: 0.9 kg CO_2 eq/kg, 2.42×10^{-2} kg CTU_e/kg.

Supplementary Fig. 1-53

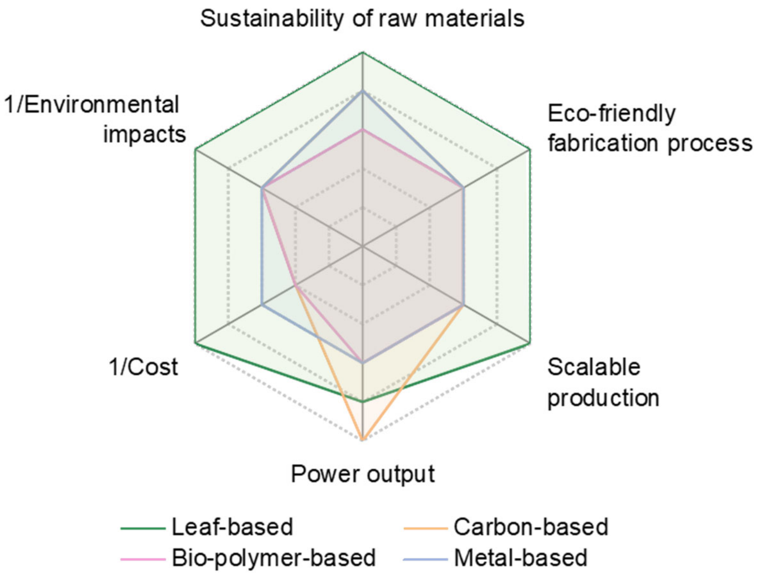

Supplementary Fig. 1 | A comparison between the LEHs of this work and previously reported other types of energy harvesters (evaluation standard in Supplementary Note 1).

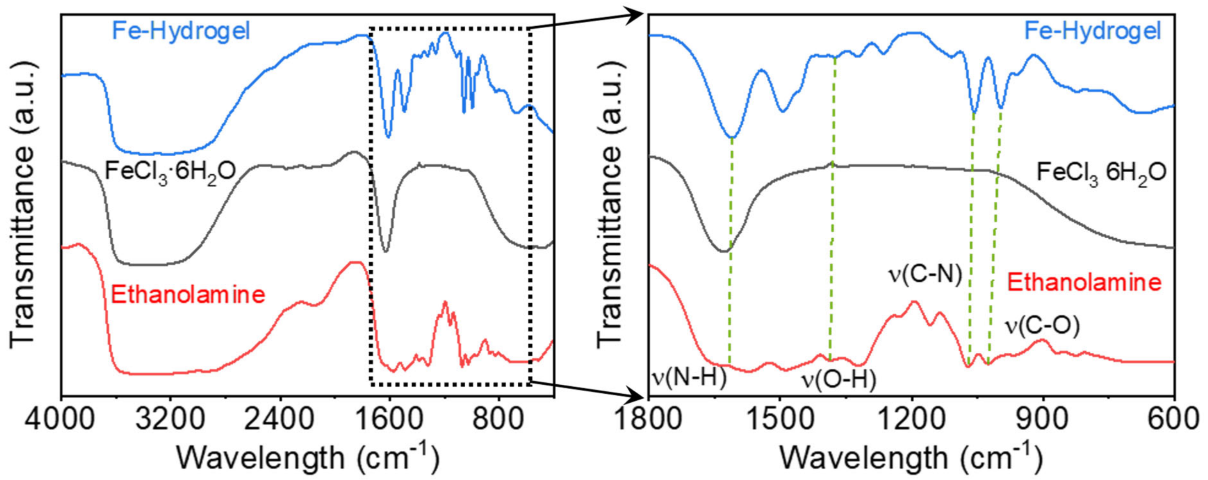

Supplementary Fig. 2 | FT-IR spectra(left), with the magnified region of interest (right) of the iron hydrogel, $FeCl_3 \cdot 6H_2O$, and EA. The redshift of ν(C-N) and ν(C-O) together with the blue shift of ν(N-H) and ν(O-H) implied successful coordination interactions between $FeCl_3 \cdot 6H_2O$ and EA.

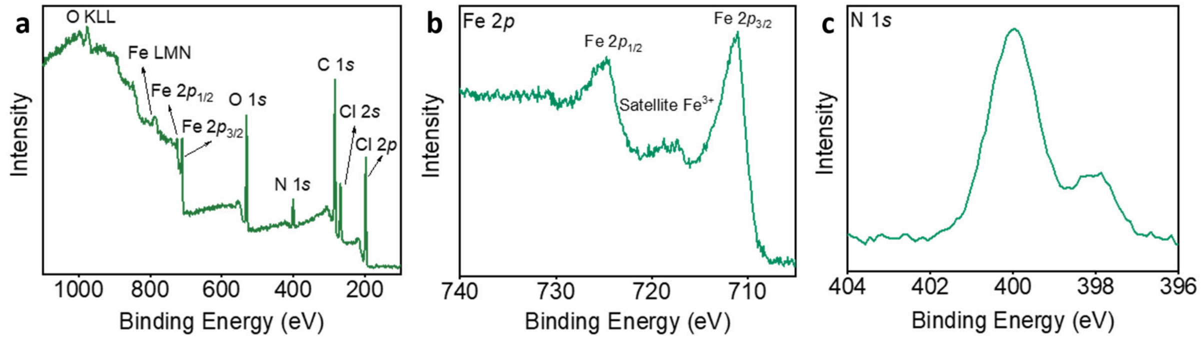

Supplementary Fig. 3 | a, XPS survey spectra of the iron hydrogel. All elements from ethanolamine and $FeCl_3 \cdot 6H_2O$ could be identified. **b**,**c**, High resolution (b) Fe 2*p* and (c) N 1*s* XPS spectra of the iron hydrogel. The presence of splitting peaks in the N 1*s* spectrum indicated a coordination interaction between EA and $FeCl_3 \cdot 6H_2O$.

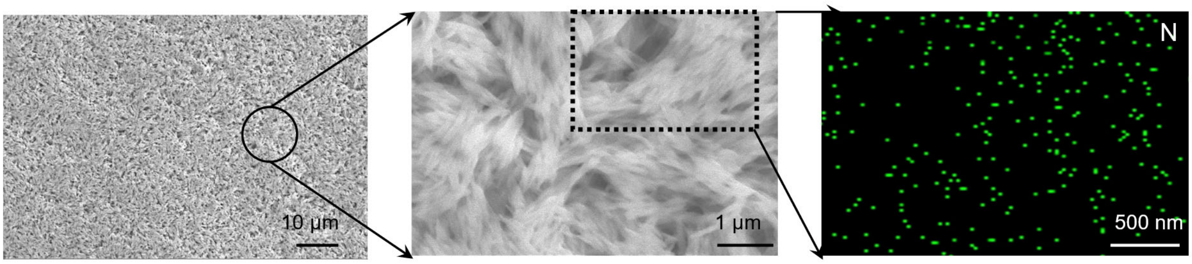

Entangled Network Structure for Fast Moisture Uptake and Release

Supplementary Fig. 4 | SEM image (left) and magnified region of interest (right) of the iron hydrogel energy-dispersive X-ray spectroscopy (EDS; right) showing the presence of N. The entangled intertwined network structure could accelerate moisture capture and vapour release.

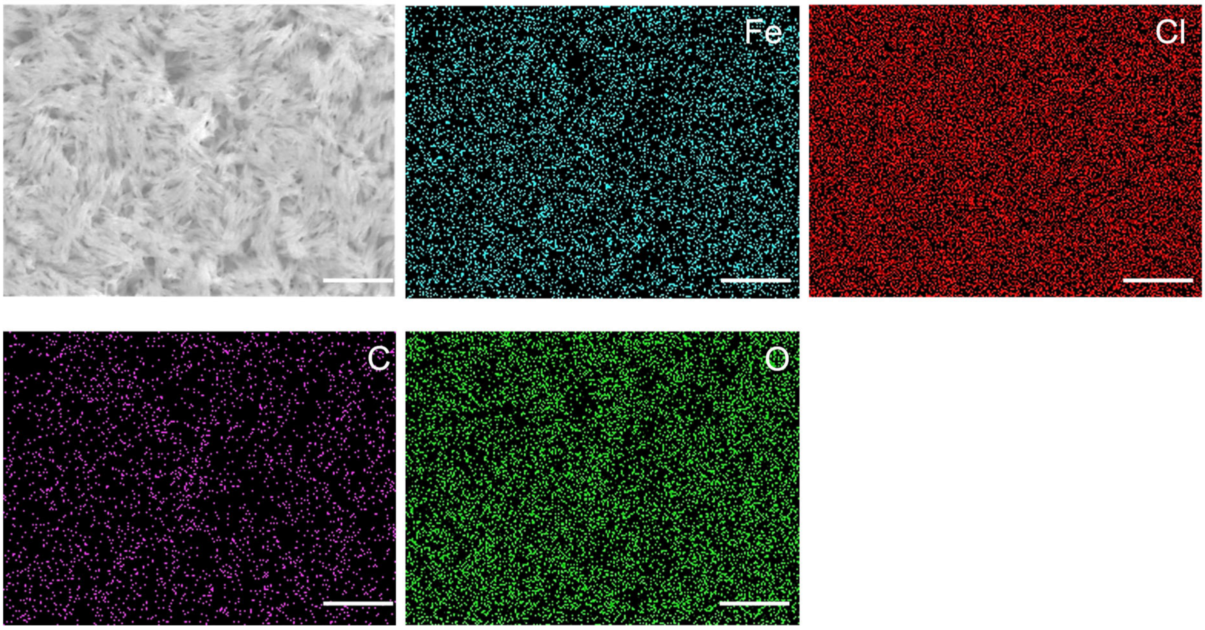

Supplementary Fig. 5 | EDS spectra of the iron hydrogel demonstrating even distribution of Fe, Cl, C, and O. Scale bar: 2.5 μm.

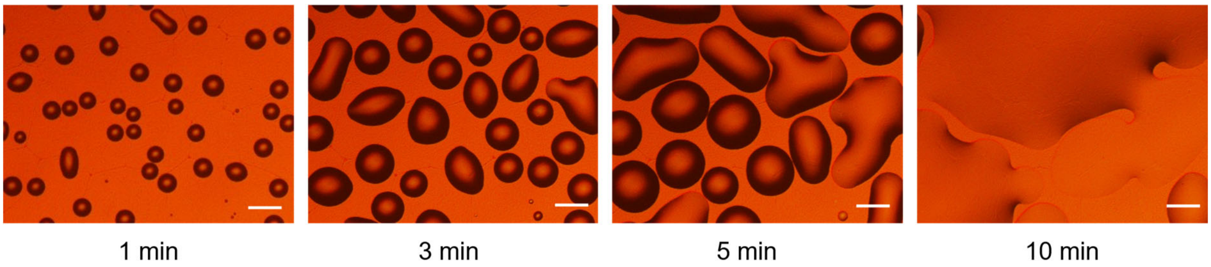

Supplementary Fig. 6 | Optical microscopy images showing the first 10 min of the moisture capture process of the iron hydrogel (25 °C, 75% RH). Large amounts of small water droplets could be viewed within 1 minute from the dehydrated iron hydrogel. Self-growth and coalescence of small droplets were then recorded within 5 min. After 10 min of moisture uptake, a macroscopic water-thin film could be viewed with the naked eye. Scale bar: 100 μm.

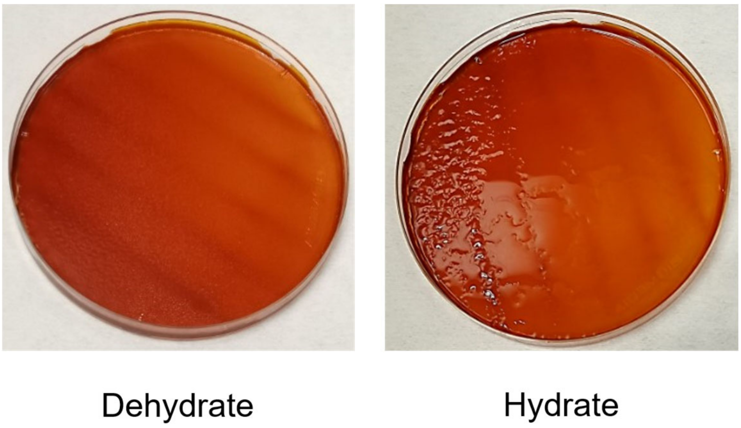

Supplementary Fig. 7 | Image of the iron hydrogel before (left) and after (right) moisture uptake. A thin water film and macroscopic droplets could be observed after 10 min of moisturization (25 °C, 75% RH).

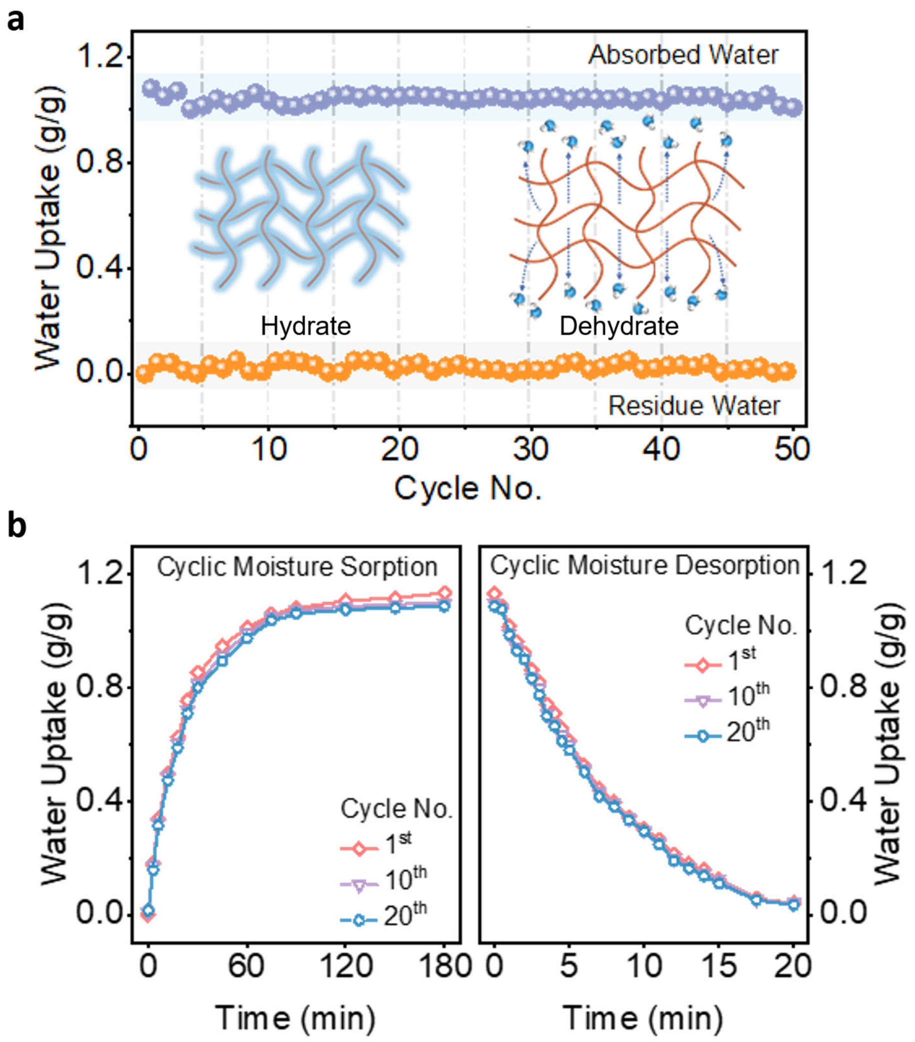

Supplementary Fig. 8 | a, Moisture absorption (~20 °C, ~78% RH) and desorption (~60 °C) cycle testing of the iron hydrogel. No significant performance reduction was observed after 50 cycles. **b**, Moisture sorption and desorption kinetics at the 1st, 10th, and 20th cycle at (~20 °C, ~80% RH).

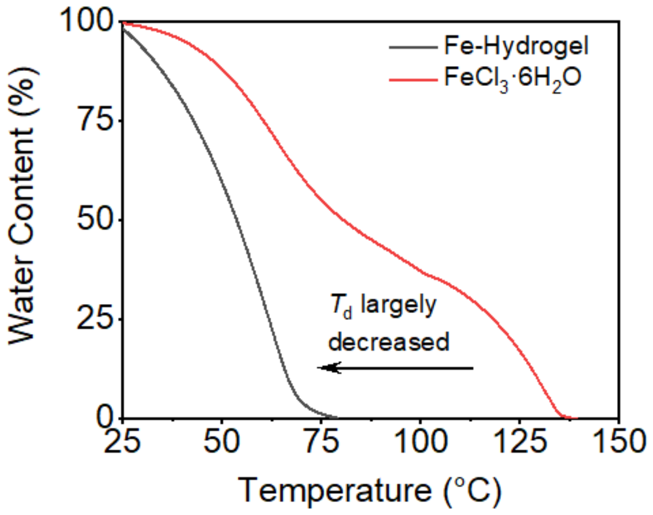

Supplementary Fig. 9 | Thermal gravimetric analysis (TGA) curves of the iron hydrogel and $FeCl_3 \cdot 6H_2O$. Samples were dehydrated and then moisturized under 75% RH 25 °C for 4 h. The desorption temperature (T_d) of the iron hydrogel decreased dramatically, which could be ascribed to the hierarchical network microscopic structure for fast vapour release. Heating rate: 2.5 °C/min.

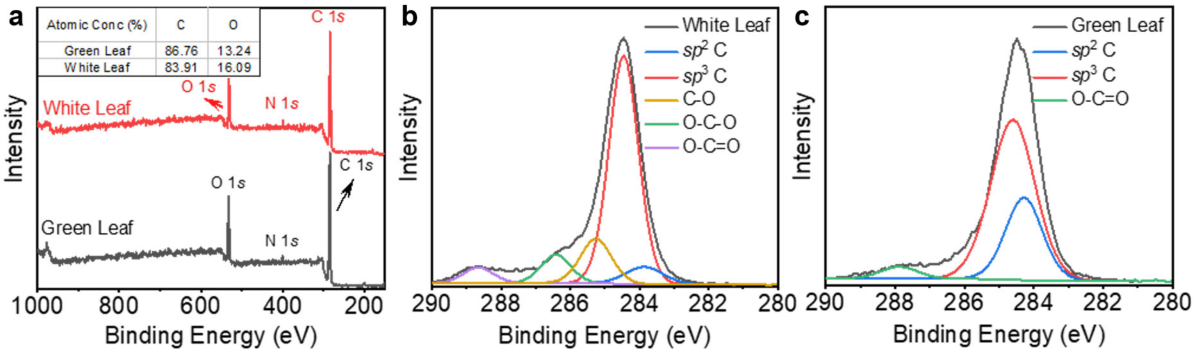

Supplementary Fig. 10 | a, XPS spectra of the green and the white leaves. **b**,**c**, High-resolution C 1*s* spectra of the (b) white and (c) green leaves.

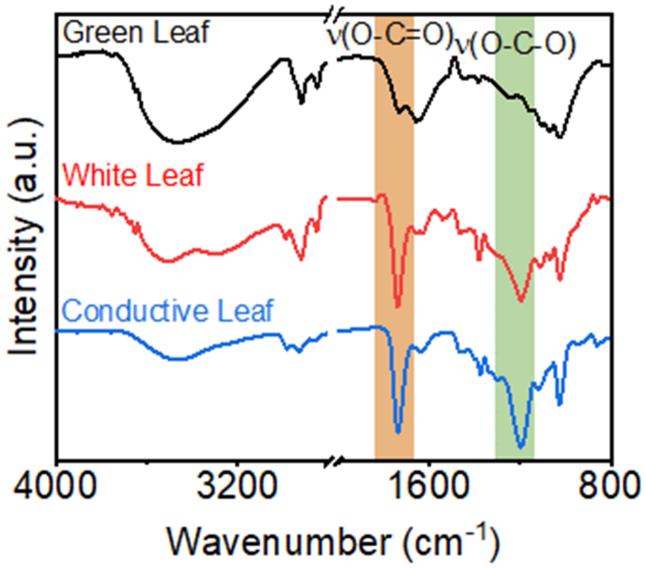

Supplementary Fig. 11 | FT-IR spectra of green, white, and conductive leaves. Enhanced C=O (1656 cm^{-1}) and C-O (1236 cm^{-1}) intensities of the white and the conductive leaf implied higher hydrophilicity after leaf bleaching.

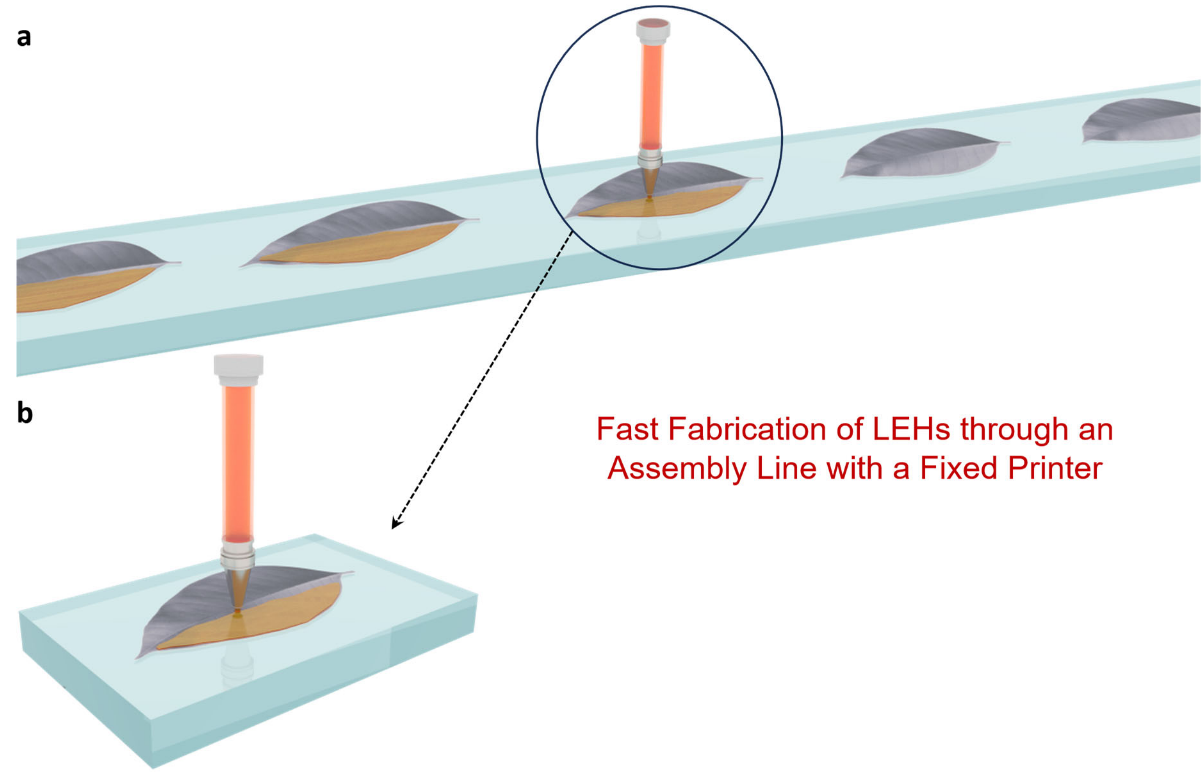

Supplementary Fig. 12 | a,**b**, Schematic illustration of a fast fabrication approach of LEHs through an assembly line with a fixed printer to precisely and pinpoint print iron hydrogel on one side of conductive leaves. For real applications, the cleaning, bleaching, and conductive treatment could be scaled up within a chemical reactor by changing different solutions or a continuous reactor with modular processing. All conductive leaves are placed on an assembly line with a printer fixed on top of it. By controlling the movement of the assembly line, the hydrogel could be automatically and concisely pinpoint coated/printed on one side of fallen leaves precisely.

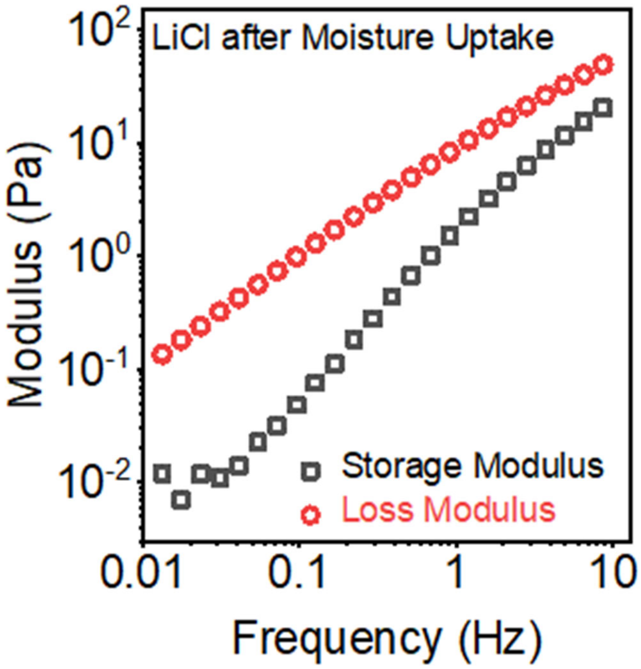

Supplementary Fig. 13 | Dynamic mechanical analysis of the LiCl under hydrate conditions. The storage modulus of the LiCl is less than the loss modulus implying the liquid-like behaviour. Therefore, the LiCl solution is easily moved across the LEH, thus damaging the asymmetrical water gradient, which is of great importance for power output.

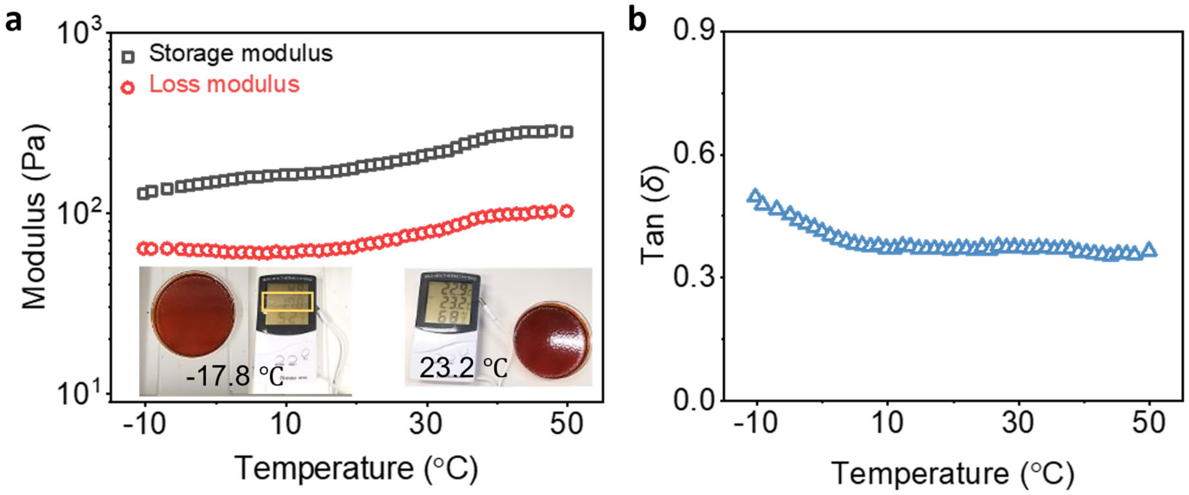

Supplementary Fig. 14 | a, Storage modulus and loss modulus of the hydrogel as a function of temperature. The strain and frequency at each temperature are set to 5% and 1 Hz, respectively. The absence of a significant increase in the storage modulus, indicates stability, confirming no freezing or significant reduction of water content under cold and hot environments. **b**, Calculated tangent (δ) from the storage and loss modulus as a function of temperature. There is no increment in the low-temperature region (-10 °C to ambient) and no reduction in the high-temperature region (ambient to 50 °C), further confirming that the hydrogel does not freeze or dry out. The slight reduction in tangent (δ) from -10 to 10 °C is attributed to the gradual evaporation of condensed water on the hydrogel surface. As the temperature drops, the environmental moisture readily condenses on the hydrogel surface, leading to more water on the gel surface and contributing to the loss modulus.

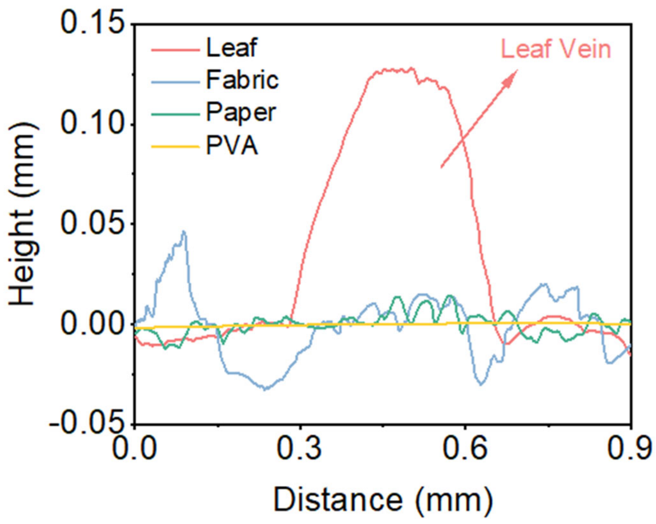

Supplementary Fig. 15 | Height measurement of different substrates through profilometer. The apparent peak represents the height of leaf veins is much higher than the surrounding mesophyll.

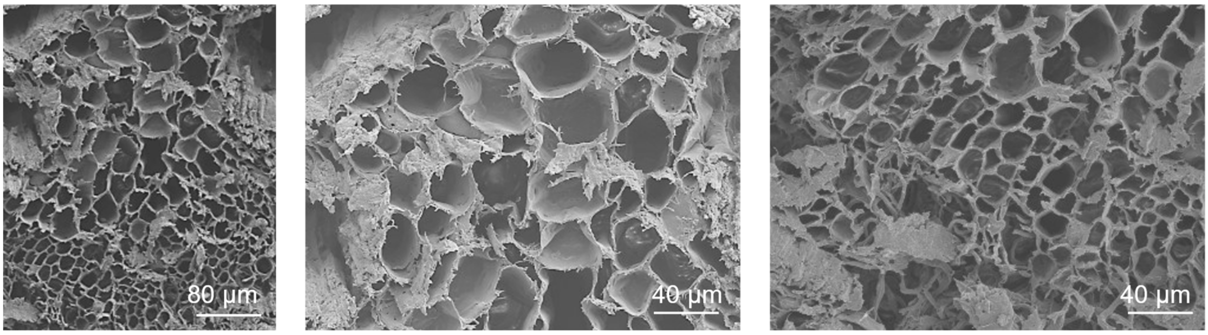

Supplementary Fig. 16 | SEM images of leaf veins. The porous structures are observed which could trap the water inside these pores when it is diffusing across the vein.

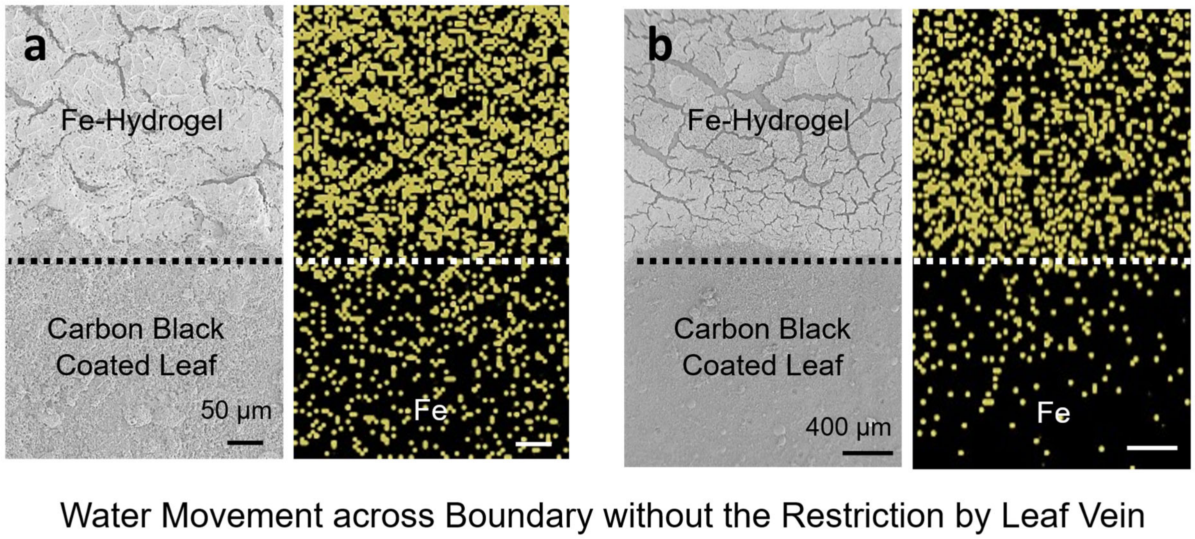

Supplementary Fig. 17 | Control experiment to highlight the importance of leaf vein on water movement restriction. SEM images and the corresponding EDX mapping of LEH without the leaf vein under different magnifications. The water movement is clearly observed by placing the sample under the ambient conditions (25 °C, 75% RH) for a day.

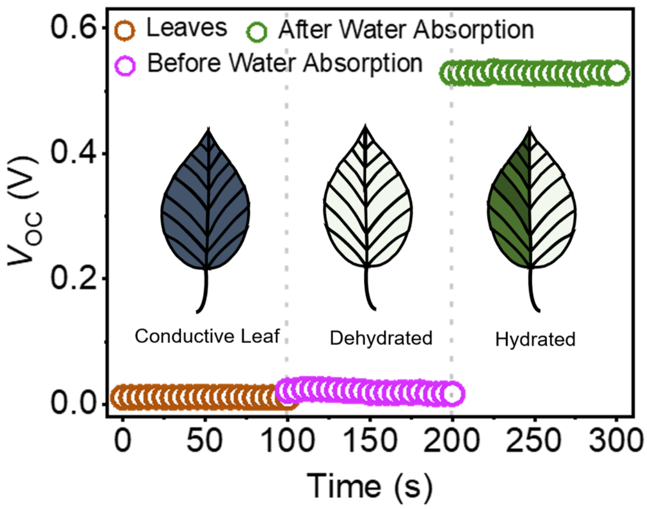

Supplementary Fig. 18 | Control experiment verifying that the potential difference was derived between the dry end and the wet end of the leaf. No electrical signal could be recorded on the conductive leaf without an iron hydrogel coating or LEH with dehydrated iron hydrogel.

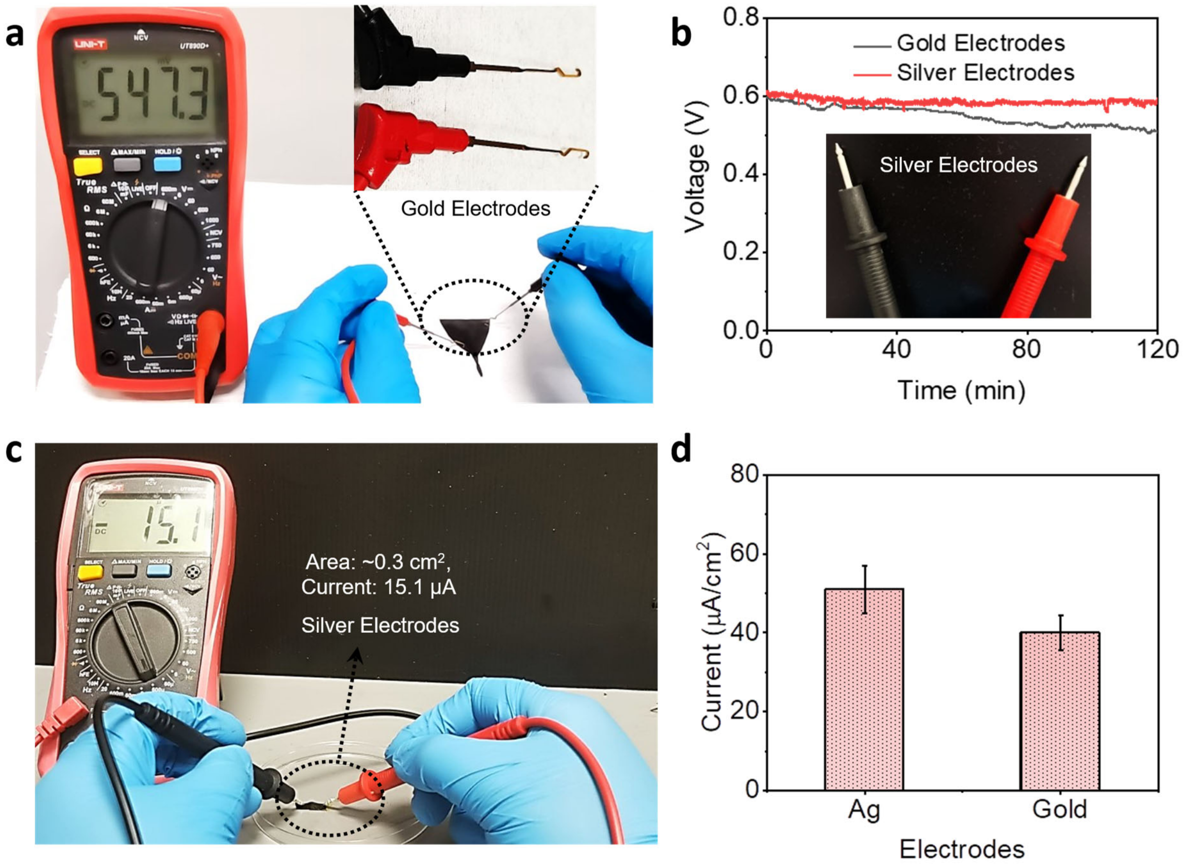

Supplementary Fig. 19 | Images of using inert electrodes for measuring the **a**, voltage, and **c**, current output of LEH. **b**, The voltage output, and **d**, the current output densities of LEH using inert electrodes (Error bar: Three-times measurement). Nearly no performance reduction is observed using inert electrodes, implying the electrode materials have fewer contributions to the observed voltage.

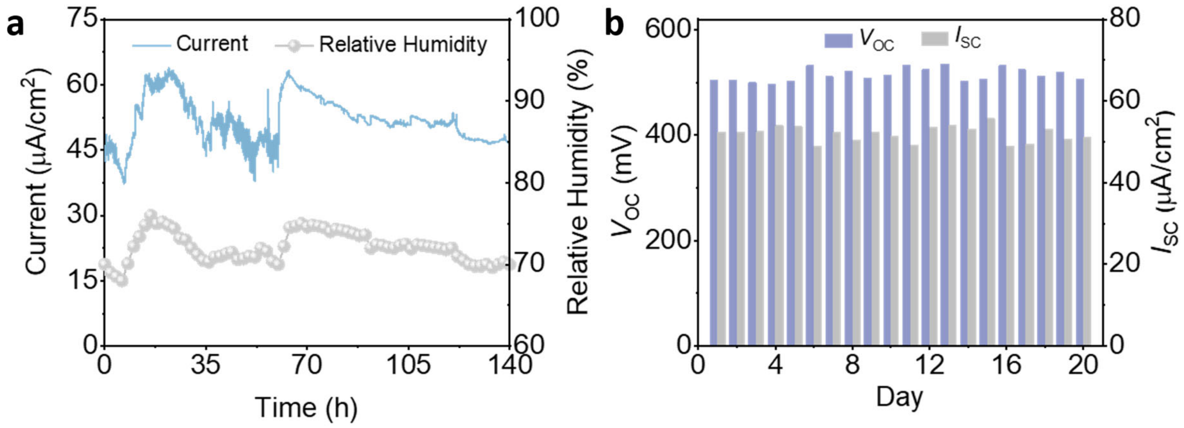

Supplementary Fig. 20 | Durability of LEH under ambient conditions. **a**, 140 h current output densities of LEH, and the corresponding relative humidity. The fluctuation of the current output densities is mainly ascribed to the relative humidity variation. **b**, Continuously monitoring voltage and current output densities for 20 days under 25 ºC and ~70-75% RH. No significant voltage, current, or mass drop is observed, implying the long-lasting water gradient is capable of sustaining the LEH power output.

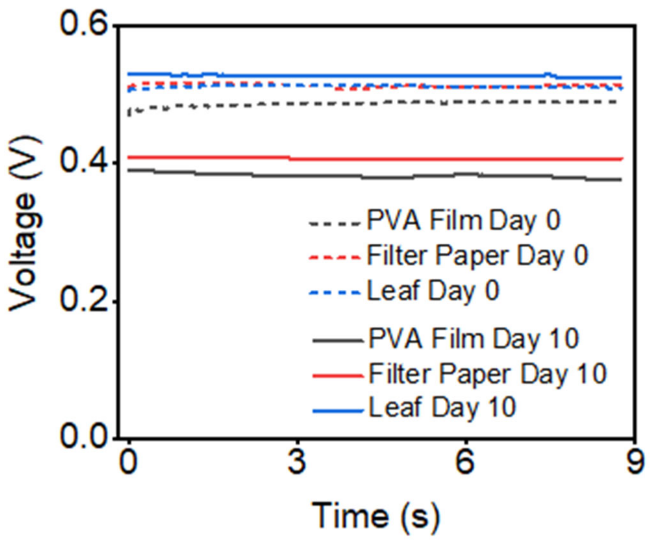

Supplementary Fig. 21 | Voltage output of energy harvesting devices with different substrates on day 0 and day 10. A voltage reduction in devices with the substrates of filter paper and PVA film is observed after 10 days, highlighting the importance of water movement cut off by the leaf vein.

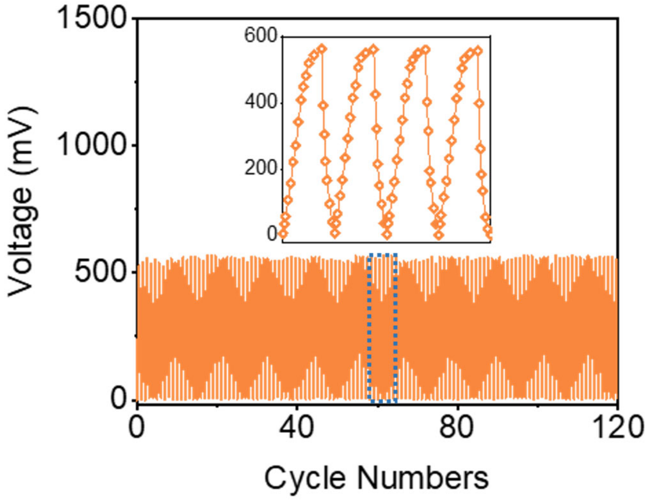

Supplementary Fig. 22 | Voltage output of LEH under cyclic moisture sorption (25 ℃, 75% RH) and desorption process (60 ℃). No performance reduction is observed for 120 cycles.

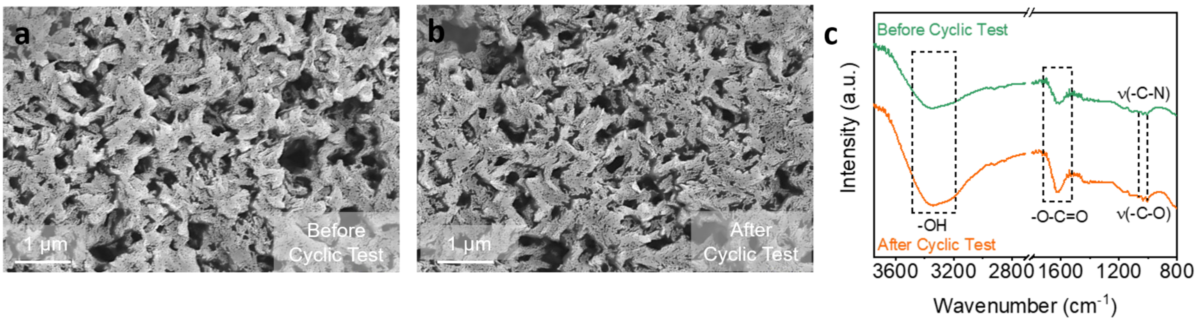

Supplementary Fig. 23 | Structure and composition analysis of Fe-Hydrogel on LEH before and after cyclic sorption and desorption process. SEM images of Fe-Hydrogel **a**, before, and **b**, after the cyclic test show no apparent morphology change. **c**, FT-IR of LEH before and after the cyclic test. There is no wavenumber shift on ν(-OH) and ν(-C-N). The strong peaks of ν(-OH) and ν(-O-C=O) are originated from the leaf.

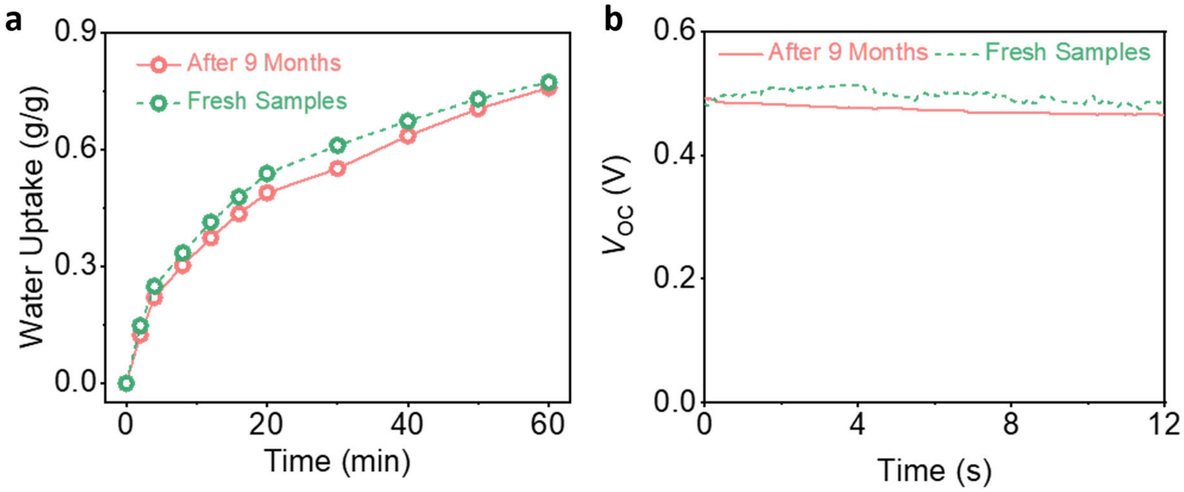

Supplementary Fig. 24 | a, Water uptake of hygroscopic hydrogel and **b**, Voltage output of LEH before and after placing in the ambient condition (25 ℃, 75% RH) for 9 months. The hydrogel still shows satisfactory water uptake, and the device could still generate ~0.5 V voltage output after 9 months, indicating the stability and robustness of hydrogel and LEH.

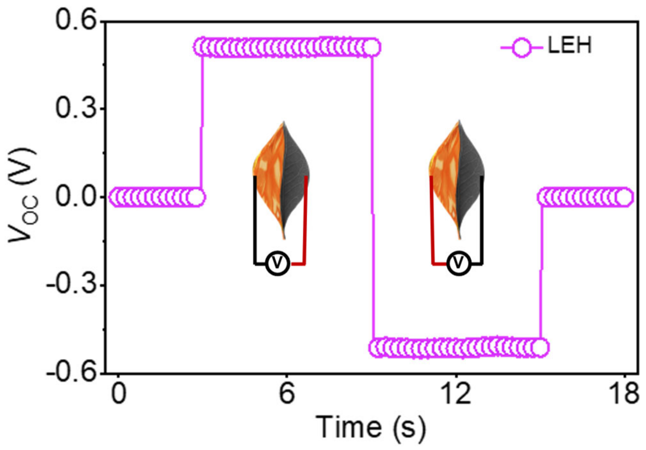

Supplementary Fig. 25 | V_{oc} measurement across the LEH based on forward and reverse directions.

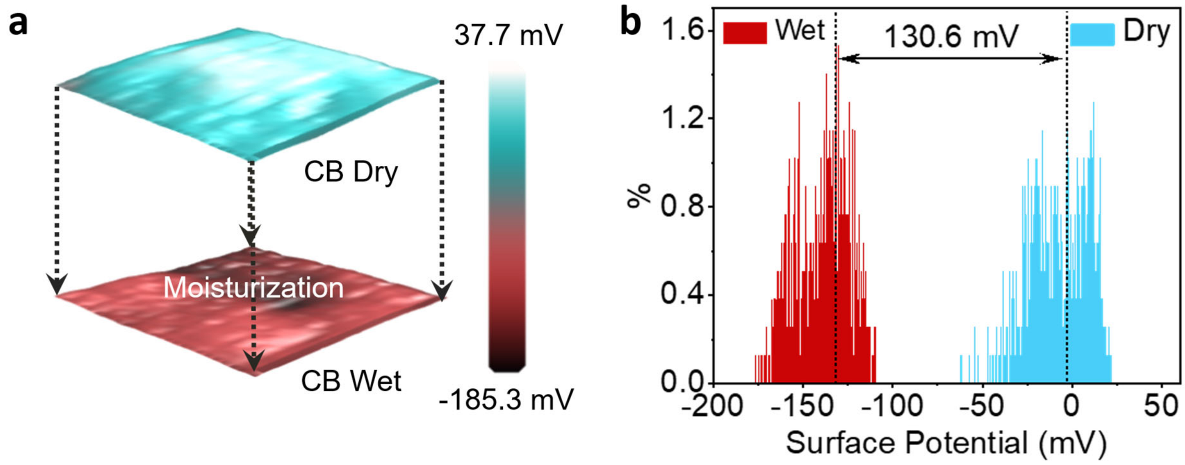

Supplementary Fig. 26 | a, KPFM images of dehydrated and slightly hydrated LEH. **b**, The Surface potential distribution of the KPFM results. A reduction in the surface potential could be observed after moisture absorption. It should be noted that the LEH is firstly dehydrated in the oven and then placed at 75% RH 25 °C. Since capturing one KPFM image requires roughly 15 minutes and low water content is required for KPFM measurement, only 130.6 mV reduction in surface potential is observed.

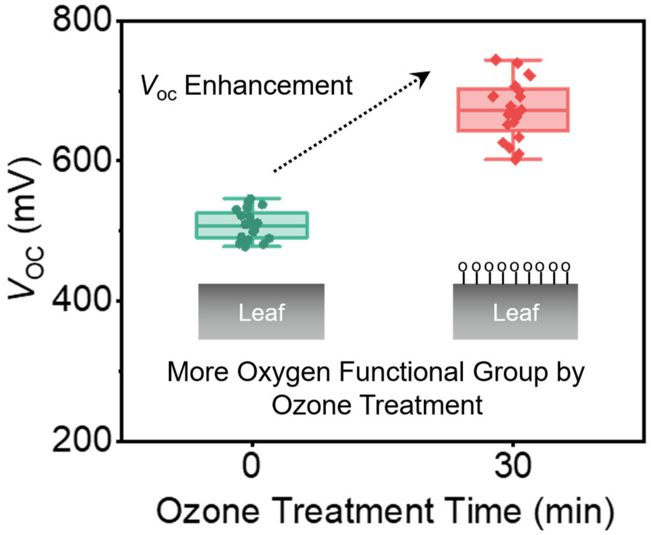

Supplementary Fig. 27 | V_{oc} of LEH as a function of ozone treatment time (Device Number: 20). After ozone treatment, more oxygen-functional groups are tethered on the leaf surface, leading to an enhancement of V_{oc} and corroborating the formation of electrical double layers (EDLs).

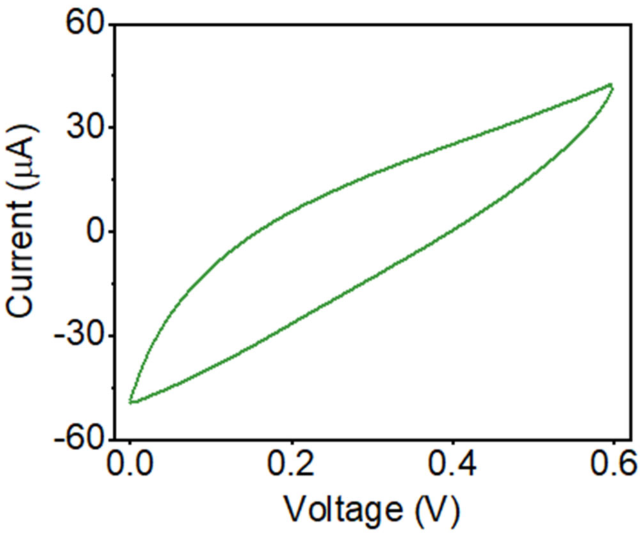

Supplementary Fig. 28 | Cyclic voltammetry (CV) curve of LEH after full moisture absorption. Scan rate: 0.05 V/s.

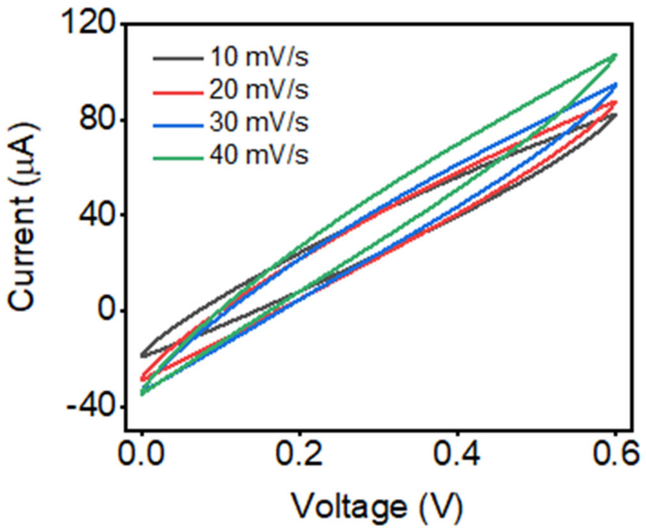

Supplementary Fig. 29 | CV curve of LEH after full moisture absorption with different scan speeds. The analogous rectangular CV shape at a low scan rate of 10 mV/s indicates that the ions adsorption process is dominated by the electric double-layer mechanism. The CV curve remains a quasi-rectangle shape and shows no significant distortion even if the scan rate is increased to 40 mV/s.

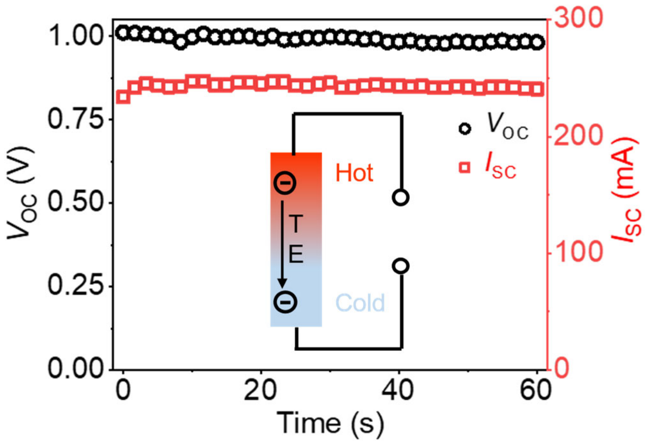

Supplementary Fig. 30 | Performance of the thermoelectric panel (TE) for charging LEH. Low-grade heat could be harvested through the TE panel.

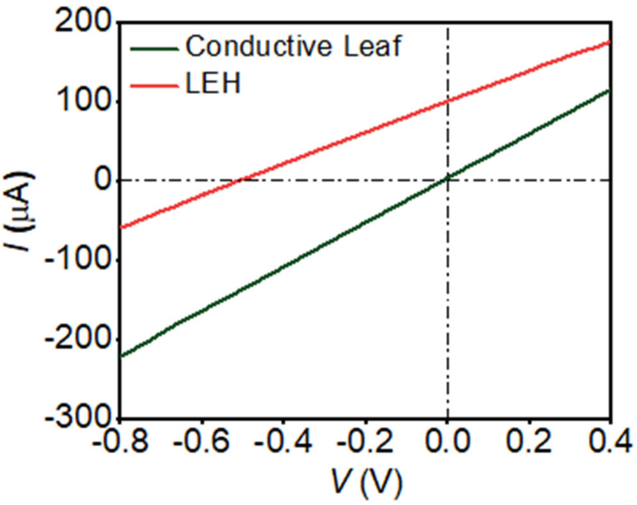

Supplementary Fig. 31 | I-V curves of a conductive leaf (black line), LEH (red line).

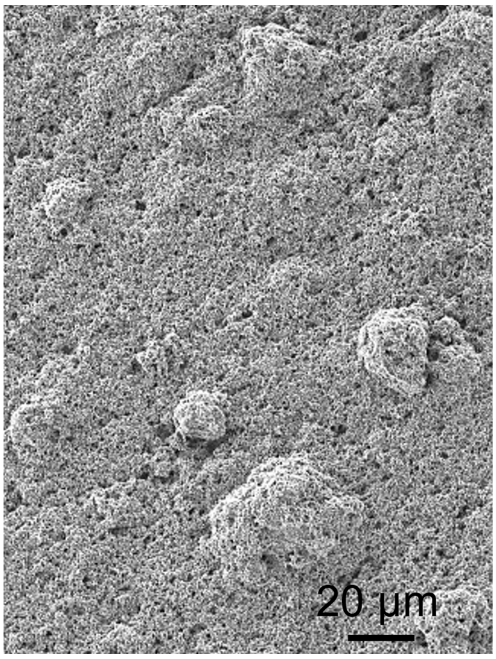

Supplementary Fig. 32 | SEM image of the PVA film after CB coating. The aggregated large CB particles could be viewed.

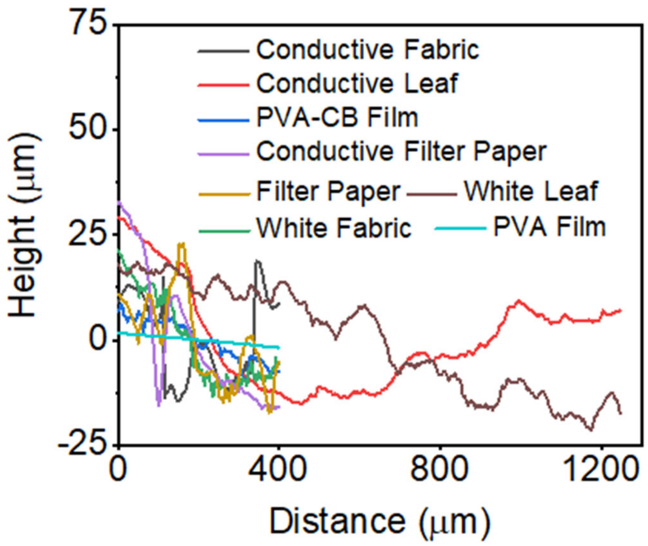

Supplementary Fig. 33 | Profilometer measurement of different substrates. The surface roughness was then calculated using these data.

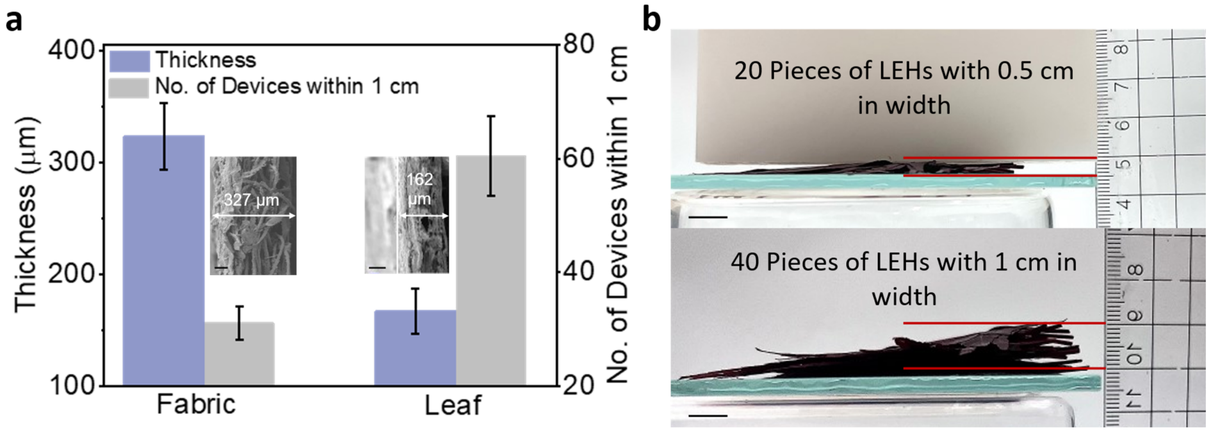

Supplementary Fig. 34 | a, Thickness comparison between the fabric and leaf. Compared with the energy harvester based on fabric, more LEH could be stacked within 1 cm due to a much lower thickness, which is beneficial for enhanced volumetric power density (Error bar: Three-times measurement). Inset: Cross-sectional SEM image of the fabric and leaf. Scale bar: 50 μm. **b**, Images showing 20 and 40 LEHs loosely and randomly stacked to heights of 0.5 cm and 1 cm heights, respectively, demonstrating the feasibility of integrating 40 LEHs within a 1 cm height in practical applications. It is important to note that the light sponge is used solely to indicate the height of the 20 LEHs, without applying any noticeable compression to the devices.

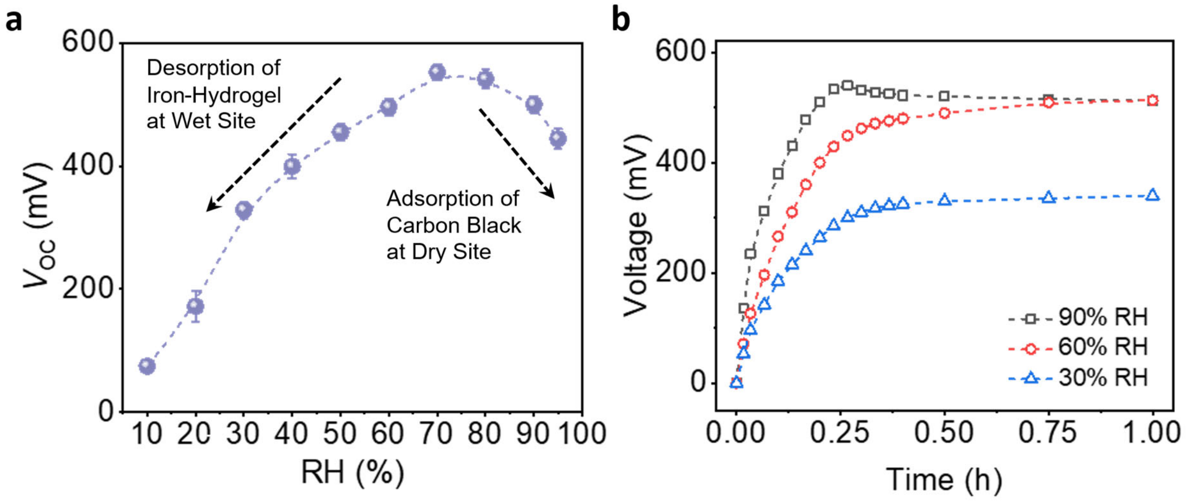

Supplementary Fig. 35 | a, Electrical performance of LEH under different RHs. The reduction of voltage output from 80% and 95% RH is ascribed to the moisture uptake of carbon black at the untreated site. All measurements are conducted at the equilibrium state. The LEH starts to demonstrate the voltage output at 20% RH and could well operate within a wide humidity range of 30-95% RH. **b**, Voltage output of initial LEH at different RH. The LEH could quickly build up voltage output within 30 minutes. The voltage output reduction of LEH at 90% RH is ascribed to the gradual moisture sorption of carbon black at the untreated sites.

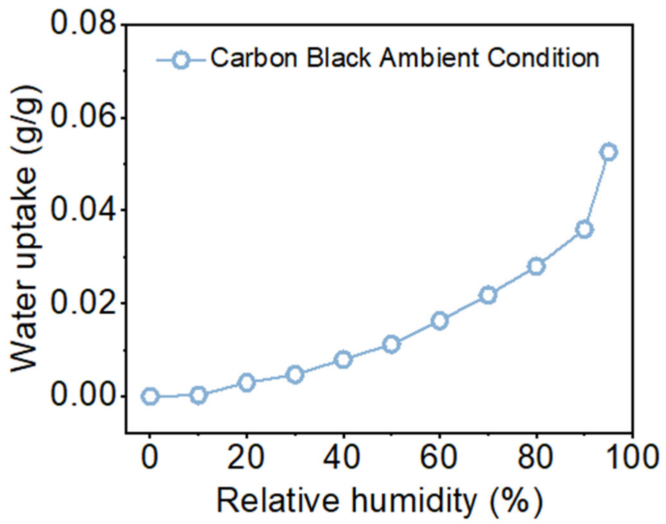

Supplementary Fig. 36 | Water uptake isotherm of carbon black under the ambient condition (25 ℃).

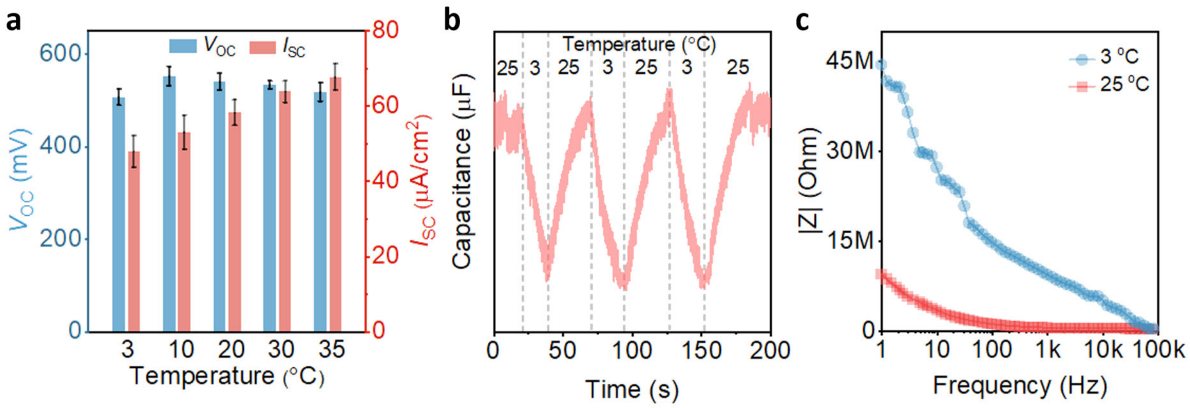

Supplementary Fig. 37 | a, LEH performance as a function of varying temperature. The humidity is controlled within 70-75% RH. An increasing trend of I_{SC} is observed with the elevated temperature, which is ascribed to **b**, the elevation of LEH capacitance with the increasing temperature that is caused by the **c**, improvement in both ionic and electrical conductivity.

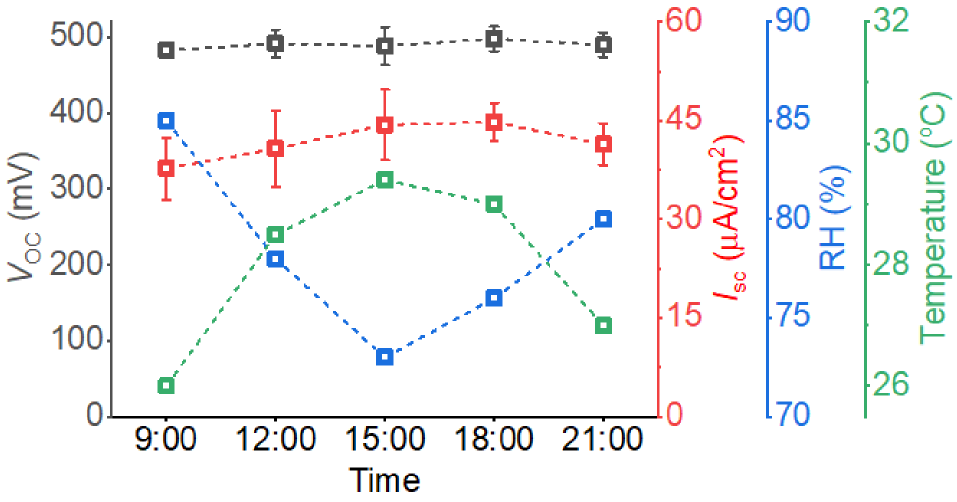

Supplementary Fig. 38 | All-day outdoor test of LEH under varying temperatures and RH (Error bar: Three-times measurement). No significant reduction in LEH performance was observed.

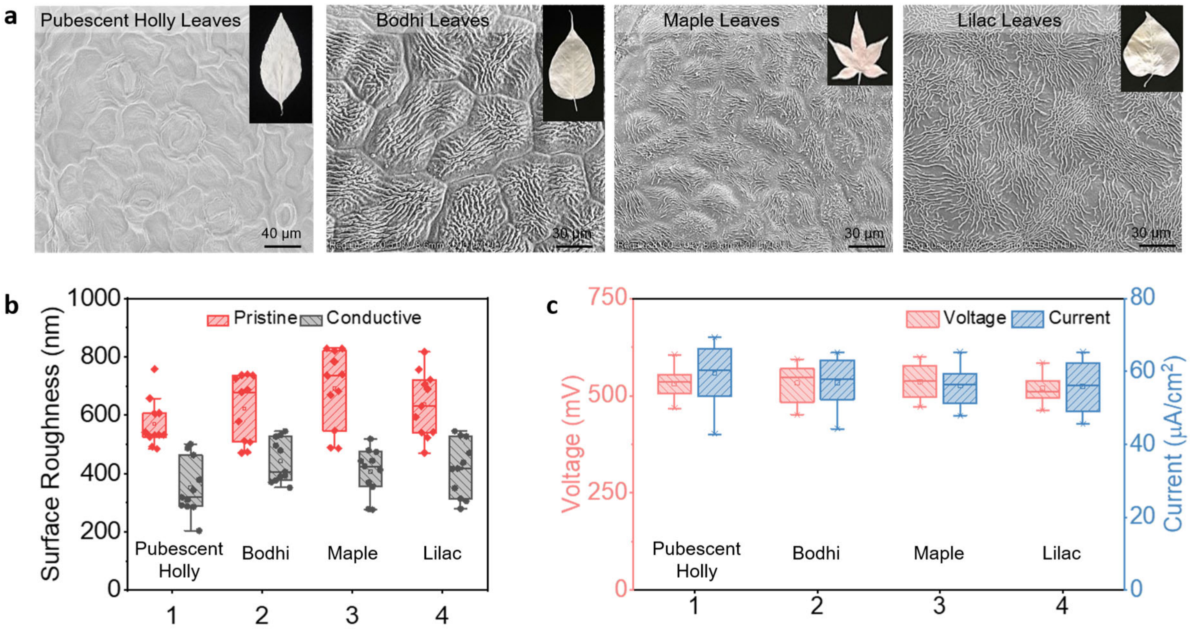

Supplementary Fig. 39 | a, SEM images of different types of fallen leaves including Pubescent Holly leaves, Bodhi leaves, Maple leaves, and Lilac leaves after bleaching treatment. The cell structures and small grooves are observed to well accommodate carbon black on their surface. **b**, Surface roughness of different kinds of white and conductive leaves calculated through profilometer measurements. (Note that the CB loading densities of different substrates were roughly the same at around 15 g/m^2). **c**, Voltage and current output densities of energy harvesters from different types of fallen leaves. Device No: 20. 1, 2, 3, 4 in **b** and **c** correspond to Pubscent Holly leaves, Bodhi leaves, Maple leaves, and Lilac leaves.

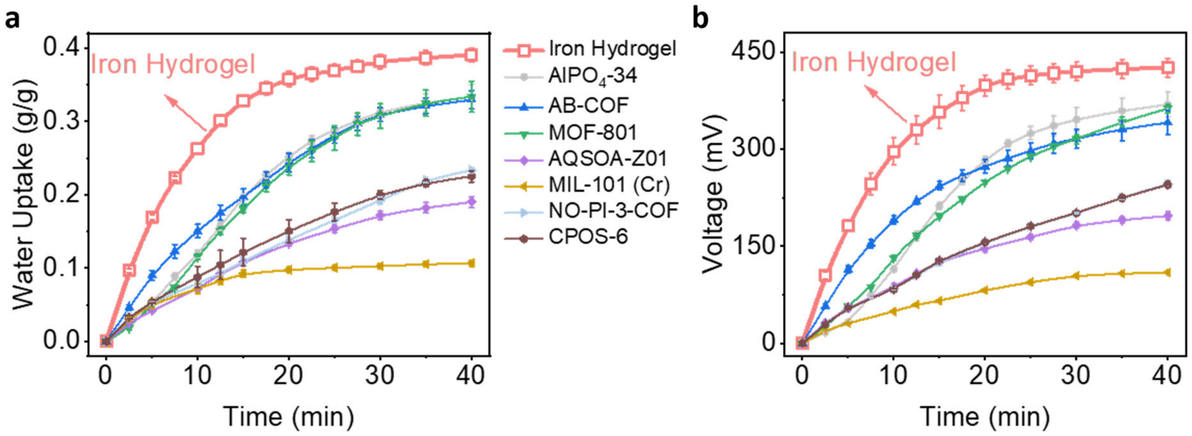

Supplementary Fig. 40 | a, Water uptake of metal and coordination-based hygroscopic materials, in comparison with iron hydrogel, and **b**, Corresponding voltage output by applying the corresponding metal and coordination-based hygroscopic materials for energy harvesting at 40% RH (Error bar: Five-times measurement). The iron hydrogel delivers better water and energy harvesting performance compared with other hygroscopic materials. Besides, the facile preparation of iron hydrogel, which merely relies on a one-step coordination reaction at room temperature is also a benefit compared with previous metal and coordination-based materials including metal-organic frameworks (MOF)[25-28] and covalent organic frameworks (COF)[29-31].

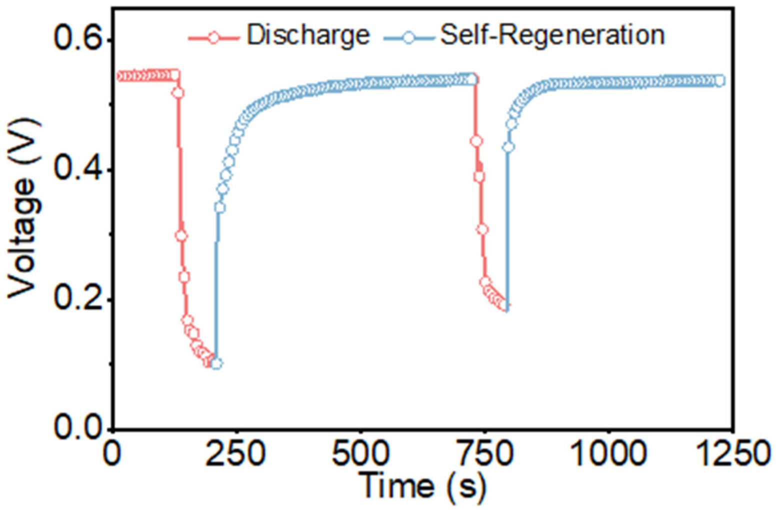

Supplementary Fig. 41 | Two times self-regeneration behaviour of LEH. V_{oc} was increased simultaneously after LEH discharge in the ambient environment.

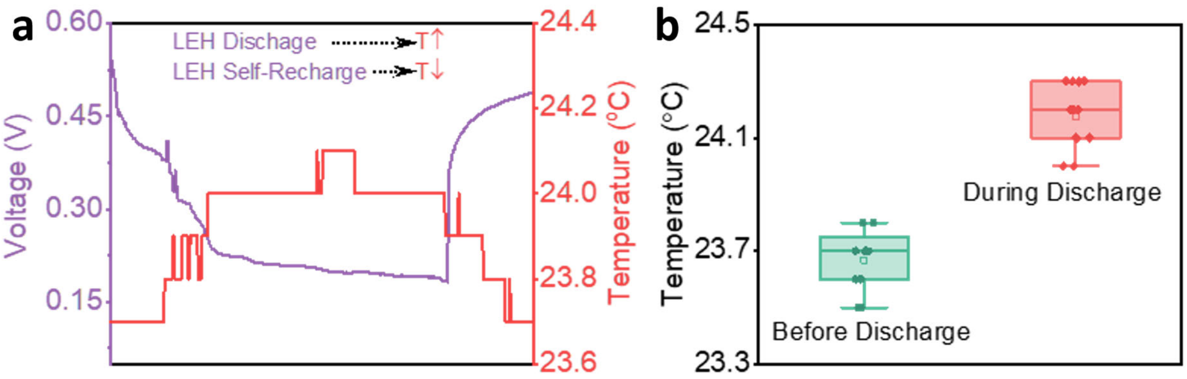

Supplementary Fig. 42 | a, Voltage and temperature change as a function of time during LEH discharge and self-recharge. **b**, Summary of the surface temperature of LEH before and during the discharge process (Device number: 12). A tiny surface temperature enhancement is monitored during the discharge process, which is strong evidence to account for the water content change during that the temperature enhancement leads to water evaporation. A temperature drop back to the initial state is also observed during the LEH self-recharge process, leading to the resorption of evaporated water by hygroscopic iron-hydrogel.

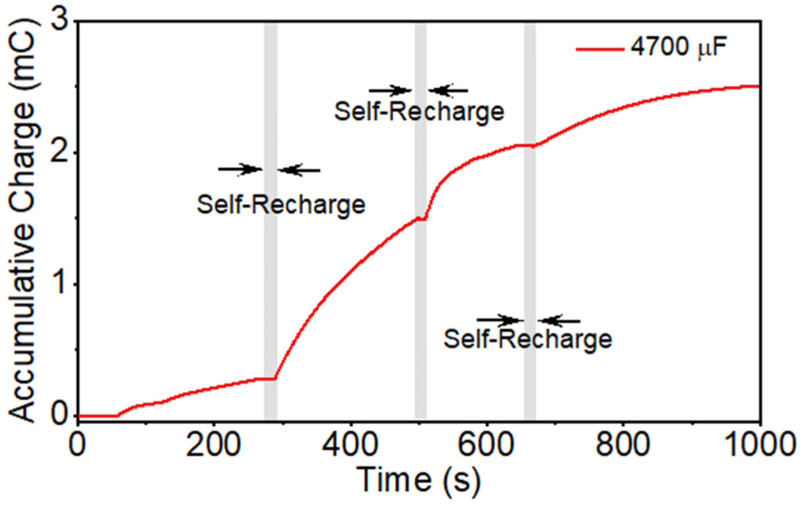

Supplementary Fig. 43 | Accumulated charge inside a 4700 μF capacitor by connecting a LEH with 4 times self-recharge, implying successive electrical output by the LEH.

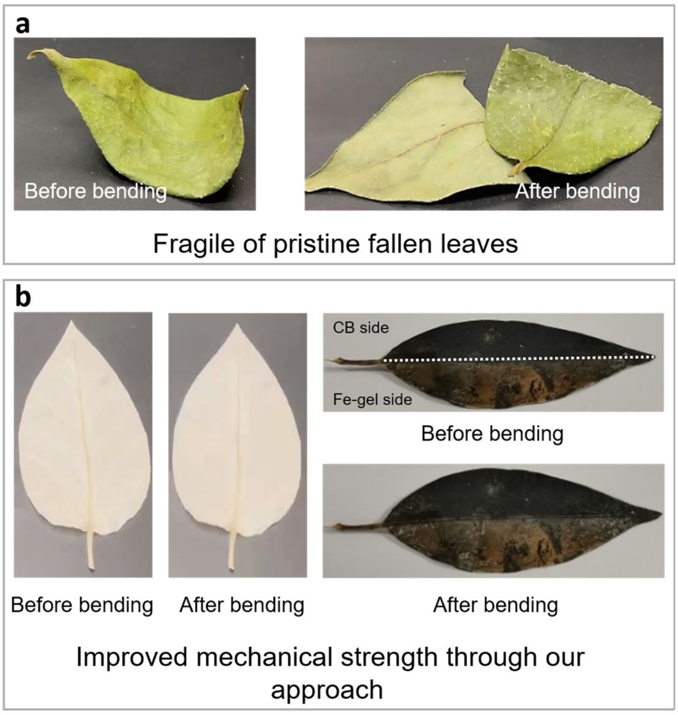

Supplementary Fig. 44 | Images of **a**, pristine fallen leaves, **b**, white leaves, and LEH through our approach after multiple bending tests. The pristine fallen leaves are very fragile, while the white leaves and LEH through our approach demonstrate flexibility with improved mechanical strength.

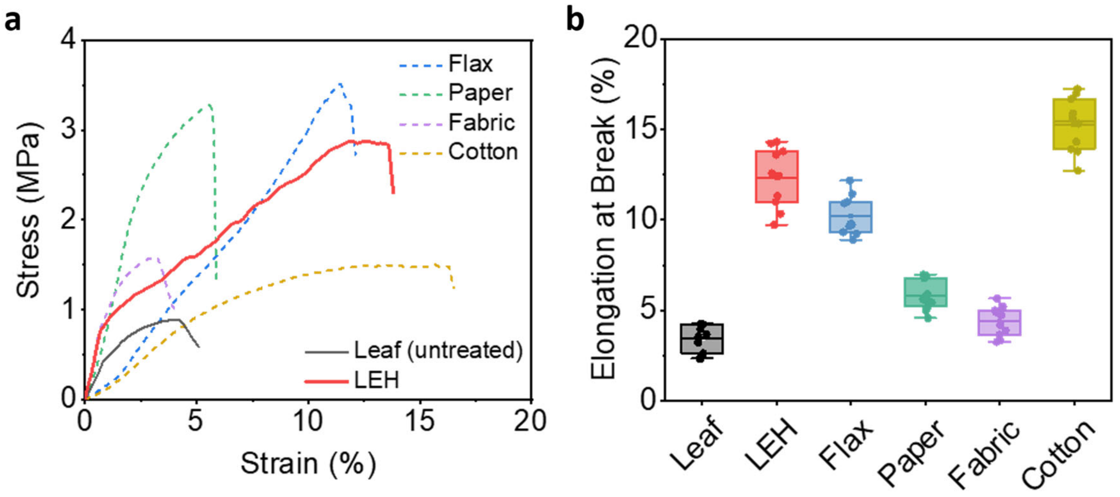

Supplementary Fig. 45 | a, stress-strain curves, **b**, elongation at break of LEH, pristine leaves, and other fiber-based materials, including flax, paper, fabric, and cotton (Device number: 10). The LEH exhibits satisfactory mechanical strength, modulus, and elongation at break compared with other fiber-based materials. The LEH also shows better mechanical properties than pristine leaves.

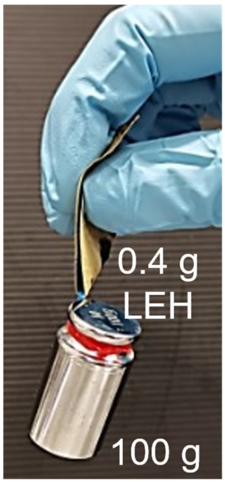

Supplementary Fig. 46 | Mechanical strength of LEH. A single device with 0.4 g could lift up a mass of 100 g without noticeable mechanical deformation.

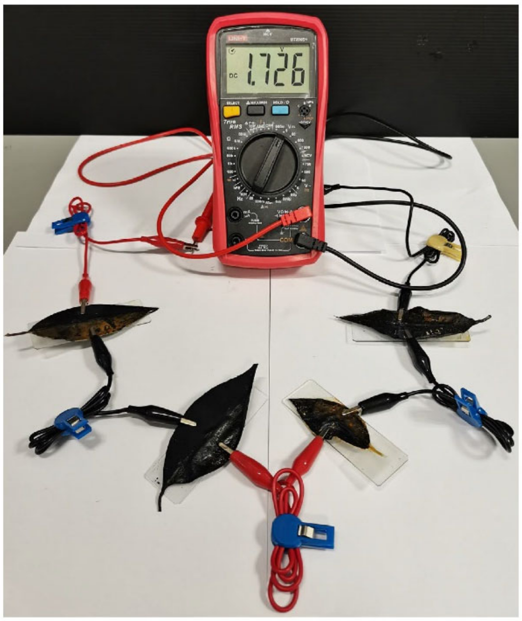

Supplementary Fig. 47 | V_{oc} output could be readily raised to 1.7 V by connecting 4 LEHs in series.

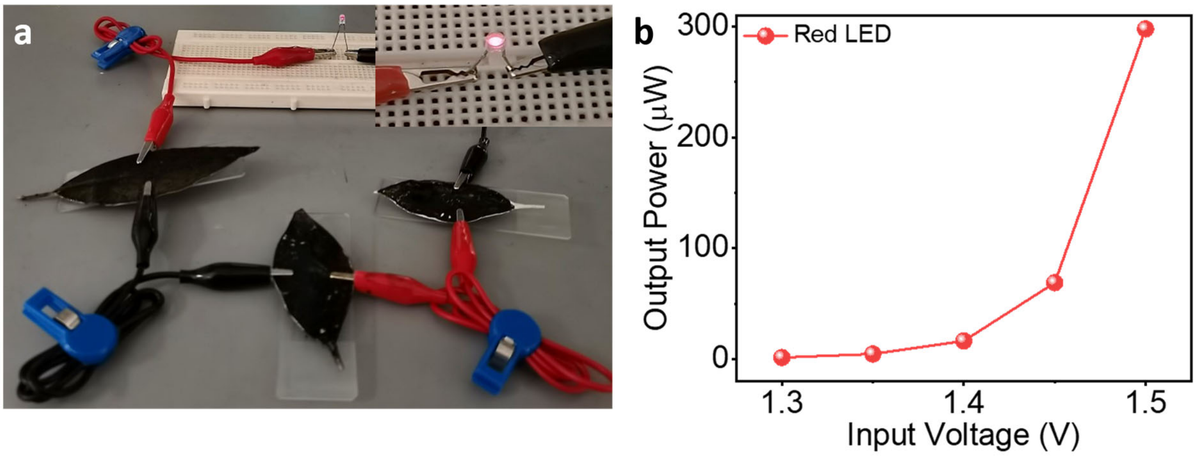

Supplementary Fig. 48 | a, Image of a powered red LED by connecting 3 LEHs in series. **b**, Power output of the red LED as the function of input voltage.

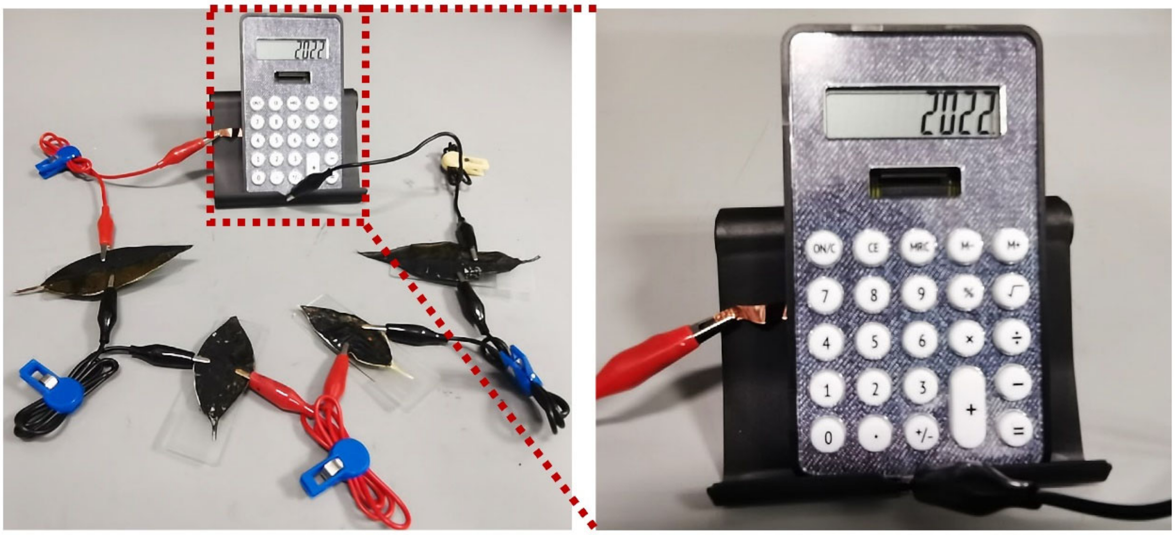

Supplementary Fig. 49 | Images of a calculator powered by connecting 4 LEHs in series.

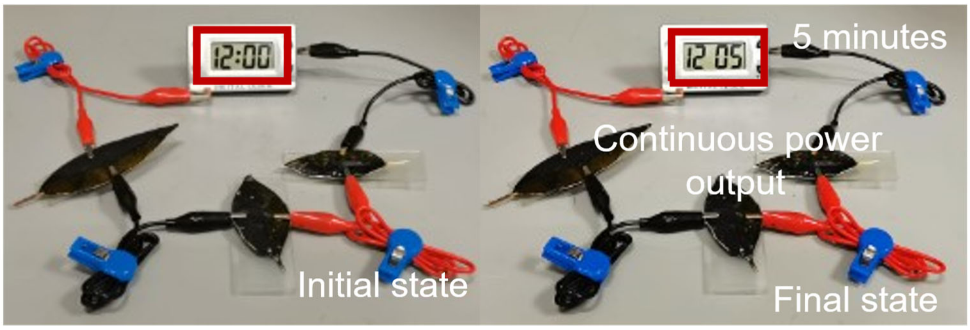

3 Connected LEHs continuously power a digital clock for 5 min

Supplementary Fig. 50 | 3 LEHs connected in series were capable of powering a digital clock for long-term display.

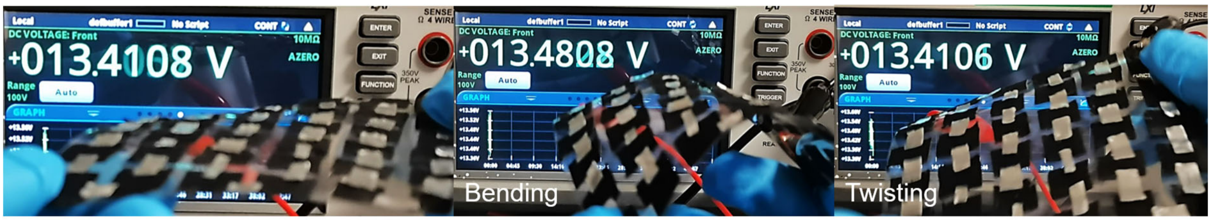

Supplementary Fig. 51 | Voltage output of LEH panel under bending and twisting conditions. The panel consists of 36 LEHs integrated on a soft polyethylene terephthalate substrate.

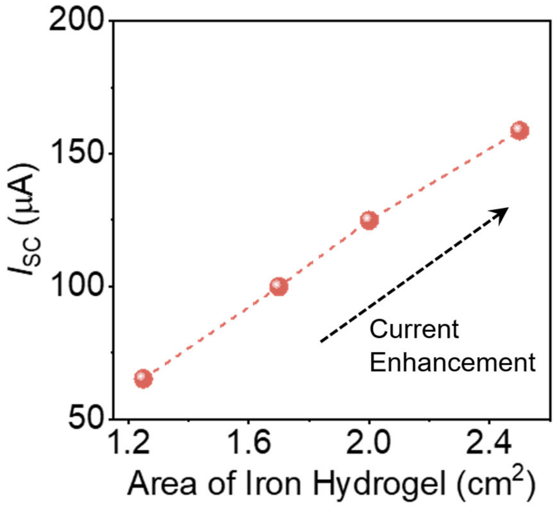

Supplementary Fig. 52 | Short circuit current (I_{sc}) as a function of the area of the iron hydrogel.

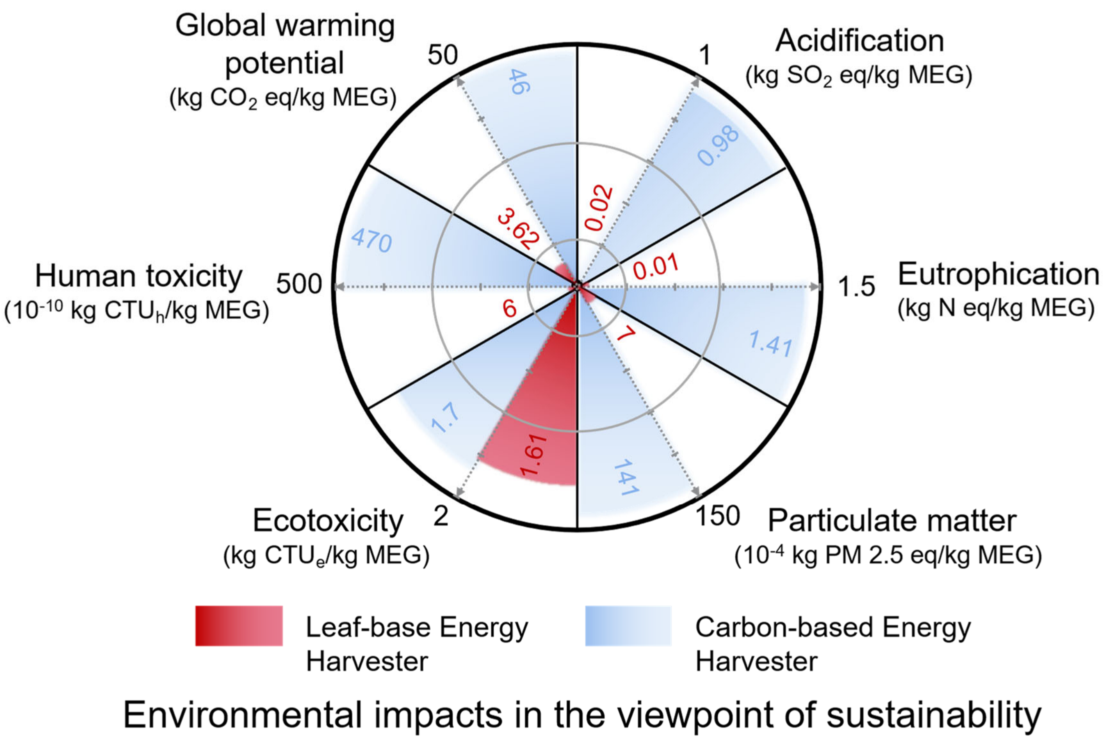

Supplementary Fig. 53 | Environmental impacts of the LEH compared with carbon-based energy harvester. CTU_e represents the comparative toxicity unit for ecotoxicity, CTU_h represents the comparative toxicity unit for human health, and PM2.5 eq represents particulate matter 2.5 equivalent.

Supplementary Tables 1-3

Supplementary Table 1 | Comparison between the LEH and other self-powered energy harvesting systems.

Energy Harvesting Materials	Mechanism	Areal Power Density	Instant or Continuous	Ref
Thermoelectric Pillar	Thermoelectric	4.5 μW/cm^2	Continuous	32
Ag_2Se Network	Thermoelectric	4 μW/cm^2	Continuous	33
ITO/PTFE/Lubricant	Water Droplet	~0.2 μW/cm^2	Instant	34
PVA Membrane	Water Droplet	~0.14 μW/cm^2	Instant	35
Ionic Hydrogel	Water Evaporation	5.1 μW/cm^2	Continuous	36
ITO/PTFE	Water Droplet	~0.56 μW/cm^2	Instant	37
PSSA/PDDA Film	Moisture	4.8 μW/cm-	Continuous	4
Protein Nanowire	Moisture	5 μW/cm^2	Continuous	2
**Leaf+Hydrogel**	**Moisture**	**12.43 μW/cm^2**	**Continuous**	**Our Work**

Supplementary Table 2 | Comparison between the LEH and other energy harvesting systems based on moisture.

Hygroscopic Materials	Areal Power Density (mW/m^2)	Output Type	Ref.
Porous carbon film	1.0×10^{-4}	Continuous	38
Cellulose	2.0×10^{-2}	Continuous	39
Protein nanowire	48.0	Continuous	2
Asymmetric ionic aerogels	3.1×10^{-3}	Continuous	40
Cationic silk nanofibrils	1.0×10^{-2}	Continuous	41
Graphite-Cellulose	2.3	Continuous	42
Cellulose Acetate	8.0×10^{-2}	Continuous	43
Whole-cell	25.0	Continuous	44
G.s-PSII	14.13	Continuous	45
3D PPy Framework	6.9	Instant	46
TiO_2 Nanowire Network	40	Instant	47
P(MEDSAH-co-AA)	11.2	Continuous	48
Paper	2.5×10^{-2}	Continuous	49
PAN Nanofiber	14.8	Continuous	50
Janus PDDA/SiNWs	22	Continuous	51
PAN Nanofiber	2.5	Continuous	52
GO/PAAS Composite	0.7	Continuous	5
Cellulon/LiCl Paper	7	Continuous	53
PSSA/PDDA Hybrid Film	55.2	Continuous	4
**Leaf+ Hydrogel**	**124.3**	**Continuous**	**Our Work**

Supplementary Table 3, Summary of water uptake and voltage output of metal and coordination-based hygroscopic materials at 40% RH.

Hygroscopic Materials	Water Uptake after 15 mins (g/g)	Water Uptake after 30 mins (g/g)	Voltage output after 30 mins (mV)	Ref.
$AlPO_4$-34	0.197	0.312	346	25
AB-COF	0.197	0.309	316	29
MOF-801	0.181	0.307	315.6	26
AQSOA-Z01	0.107	0.171	182.3	27
MIL-101 (Cr)	0.092	0.102	104.3	28
NO-PI-3-COF	0.109	0.192	202.2	30
CPOS-6	0.121	0.20	201.2	31
**Iron Hydrogel**	**0.328**	**0.381**	**420.5**	**Our Work**

Supplementary References

1. Shen, D. et al. Self-Powered Wearable Electronics Based on Moisture Enabled Electricity Generation. *Adv. Mater.* **30**, e1705925 (2018).

2. Liu, X. et al. Power generation from ambient humidity using protein nanowires. *Nature* **578**, 550-554 (2020).

3. Liu, X., Gao. H., Sun. L., Yao, J. Generic Air-Gen Effect in Nanoporous Materials for Sustainable Energy Harvesting from Air Humidity. *Adv. Mater.* **36**, e2300748 (2024).

4. Wang, H. et al. Bilayer of polyelectrolyte films for spontaneous power generation in air up to an integrated 1,000 V output. *Nat. Nanotechnol.* **16**, 811-819 (2021).

5. Huang, Y. et al. All-region-applicable, continuous power supply of graphene oxide composite. *Energy Environ. Sci.* **12**, 1848-1856 (2019).

6. Xu, W. et al. A droplet-based electricity generator with high instantaneous power density. *Nature* **578**, 392-396 (2020).

7. Yang, Y. et al. A laser-engraved wearable sensor for sensitive detection of uric acid and tyrosine in sweat. *Nat. Biotechnol.* **38**, 217-224 (2020).

8. Lu, Y. et al. Stretchable graphene–hydrogel interfaces for wearable and implantable bioelectronics. *Nat. Electron.* **7**, 51-65 (2024).

9. Kresse, G. & Furthmüller, J. Efficient iterative schemes for ab initio total-energy calculations using a plane-wave basis set. *Phys. Rev. B* **54**, 11169-11186 (1996).

10. Perdew, J. P., Burke, K. & Ernzerhof, M. Generalized Gradient Approximation Made Simple. *Phys. Rev. Lett.* **77**, 3865-3868 (1996).

11. Blochl, P. E. Projector augmented-wave method. *Phys. Rev. B* **50**, 17953-17979 (1994).

12. Monkhorst, H. J. & Pack, J. D. Special points for Brillouin-zone integrations. *Phys. Rev. B* **13**, 5188-5192 (1976).

13. Abdelhak, J., Cherni, S. N., Zid, M. F. & Driss, A. Crystal structure, spectroscopic and magnetic properties of a new iron(III) complex. *J. Struct. Chem.* **56**, 654-661 (2015).

14. Yang, J. et al. A Moisture-Hungry Copper Complex Harvesting Air Moisture for Potable Water and Autonomous Urban Agriculture. *Adv. Mater.* **32**, e2002936 (2020).

15. Yamashita, T. & Hayes, P. Analysis of XPS spectra of Fe^{2+} and Fe^{3+} ions in oxide materials.

Appl. Surf. Sci. **254**, 2441-2449 (2008).

16. Zhang, X. et al. Super-hygroscopic film for wearables with dual functions of expediting sweat evaporation and energy harvesting. *Nano Energy* **75**, 104873 (2020).

17. Zhai, S. et al. Enhanced Proton Conductivity in Sulfonated Poly(ether ether ketone) Membranes by Incorporating Sodium Dodecyl Benzene Sulfonate. *Polymers* **11**, 203 (2019).

18. Weidema, B. P. et al. The ecoinvent database: Overview and methodology, Data quality guideline for the ecoinvent database version 3. (2013)

19. Turk, J. et al. Evaluation of an environmental profile comparison for nanocellulose production and supply chain by applying different life cycle assessment methods. *J. Cleaner Prod.* **247**, 119107 (2020).

20. Rai, R., Ranjan, R. & Dhar, P. Life cycle assessment of transparent wood production using emerging technologies and strategic scale-up framework. *Sci. Total Environ.* **846**, 157301 (2022).

21. Li, Q., McGinnis, S., Sydnor, C., Wong, A. & Renneckar, S. Nanocellulose Life Cycle Assessment. *ACS Sustainable Chem. Eng.* **1**, 919-928 (2013).

22. Bare, J. TRACI 2.0: the tool for the reduction and assessment of chemical and other environmental impacts 2.0. *Clean Technol. Environ. Policy* **13**, 687-696 (2011).

23. Arvidsson, R., Kushnir, D., Sanden, B. A. & Molander, S. Prospective life cycle assessment of graphene production by ultrasonication and chemical reduction. *Environ. Sci. Technol.* **48**, 4529-4536 (2014).

24. Serrano-Luján, L. et al. Environmental impact of the production of graphene oxide and reduced graphene oxide. *SN Appl. Sci.* **1**, 179 (2019).

25. Krajnc A, Varlec J, Mazaj M, Ristić A, Logar NZ, Mali G. Superior Performance of Microporous Aluminophosphate with LTA Topology in Solar-Energy Storage and Heat Reallocation. *Adv. Energy Mater.* **7**, 1601815 (2017).

26. Kim H*, et al.* Water harvesting from air with metal-organic frameworks powered by natural sunlight. *Science* **356**,430-434(2017).

27. LaPotin A*, et al.* Dual-Stage Atmospheric Water Harvesting Device for Scalable Solar-Driven Water Production. *Joule* **5**, 166-182 (2021).

28. Xu W, Yaghi OM. Metal–Organic Frameworks for Water Harvesting from Air, Anywhere,

Anytime. *ACS Cent. Sci.* **6**, 1348-1354 (2020).

29. Nguyen HL, Gropp C, Hanikel N, Mockel A, Lund A, Yaghi OM. Hydrazine-Hydrazide-Linked Covalent Organic Frameworks for Water Harvesting. *ACS Cent. Sci.* **8**, 926-932 (2022).

30. Grunenberg L*, et al.* Postsynthetic Transformation of Imine- into Nitrone-Linked Covalent Organic Frameworks for Atmospheric Water Harvesting at Decreased Humidity. *J. Am. Chem. Soc.* **145**, 13241-13248 (2023).

31. Zhang S, Fu J, Das S, Ye K, Zhu W, Ben T. Crystalline Porous Organic Salt for Ultrarapid Adsorption/Desorption-Based Atmospheric Water Harvesting by Dual Hydrogen Bond System. *Angew Chem. Int. Ed.* **61**, e202208660 (2022).

32. Hong, S. et al. Wearable thermoelectrics for personalized thermoregulation. *Sci. Adv.* **5**, eaaw0536 (2019).

33. Liu, Y. et al. Scalable-produced 3D elastic thermoelectric network for body heat harvesting. *Nat. Commun.* **14**, 3058 (2023).

34. Xu, W. et al. SLIPS-TENG: robust triboelectric nanogenerator with optical and charge transparency using a slippery interface. *Natl. Sci. Rev.* **6**, 540-550 (2019).

35. Nie, J., Wang, Z., Ren, Z., Li, S., Chen, X., Wang, Z. Power generation from the interaction of a liquid droplet and a liquid membrane. *Nat. Commun.* **10**, 2264 (2019).

36. Sun, Z. et al. Achieving efficient power generation by designing bioinspired and multi-layered interfacial evaporator. *Nat. Commun.* **13**, 5077 (2022).

37. Yan, X. et al. Bubble energy generator. *Sci. Adv.* **8**, eabo7698 (2022).

38. Liu, K. *et al.* Induced potential in porous carbon films through water vapor absorption. *Angew Chem. Int. Ed.* **55**, 8003-8007 (2016).

39. Li, M. *et al.* Biological Nanofibrous Generator for Electricity Harvest from Moist Air Flow. *Adv. Funct. Mater.* **29**, 1901798 (2019).

40. Yang, W., Lv, L., Li, X., Han, X., Li, M., Li, C. Quaternized silk nanofibrils for electricity generation from moisture and ion rectification. *ACS Nano* **14**, 10600-10607 (2020).

41. Moreira, K.S., Lermen, D., dos Santos, L.P., Galembeck, F., Burgo, T.A. Flexible, low-cost and scalable, nanostructured conductive paper-based, efficient hygroelectric generator. *Energy Environ. Sci.* **14**, 353-358 (2021).

42. Lyu, Q., Peng, B., Xie, Z., Du, S., Zhang, L., Zhu, J. Moist-induced electricity generation

by electrospun cellulose acetate membranes with optimized porous structures. *ACS Appl Mater Inter* **12**, 57373-57381 (2020).

43. Yang, W. *et al.* Asymmetric ionic aerogel of biologic nanofibrils for harvesting electricity from moisture. *Nano Energy* **71**, 104610 (2020).

44. Ren, G. *et al.* A facile and sustainable hygroelectric generator using whole-cell Geobacter sulfurreducens. *Nano Energy* **89**, 106361 (2021).

45. Ren, G, Hu, Q, Ye, J, Hu, A, Lv, J, Zhou, S. All-Biobased Hydrovoltaic-Photovoltaic Electricity Generators for All-Weather Energy Harvesting. *Research* (2022), DOI: 10.34133/2022/9873203.

46. Xue, J. et al. Vapor-Activated Power Generation on Conductive Polymer. *Adv. Funct. Mater.* **26**, 8784-8792 (2016).

47. Shen, D. et al. Self-Powered Wearable Electronics Based on Moisture Enabled Electricity Generation. *Adv. Mater.* **30**, e1705925 (2018).

48. Long, Y. et al. Moisture-induced autonomous surface potential oscillations for energy harvesting. *Nat. Commun.* **12**, 5287 (2021).

49. Gao, X. et al. Electric power generation using paper materials. *J. Mater. Chem. A* **7**, 20574-20578 (2019).

50. Sun, Z. et al. Nanofiber fabric based ion-gradient-enhanced moist-electric generator with a sustained voltage output of 1.1 volts. *Mater. Horiz.* **8**, 2303-2309 (2021).

51. Wu, Y. et al. A Hygroscopic Janus Heterojunction for Continuous Moisture-Triggered Electricity Generators. *ACS Appl. Mater. Interfaces* **14**, 19569-19578 (2022).

52. Sun, Z., Wen, X., Wang, L., Yu, J. & Qin, X. Capacitor-inspired high-performance and durable moist-electric generator. *Energy Environ. Sci.* **15**, 4584-4591 (2022).

53. Tan, J. et al. Self-sustained electricity generator driven by the compatible integration of ambient moisture adsorption and evaporation. *Nat. Commun.* **13**, 3643 (2022).
