# Supplementary material for: Leaf-based energy harvesting and storage utilizing hygroscopic iron hydrogel for continuous power generation
Source: Nat Commun. 2025 Jun 6;16:5267. doi: 10.1038/s41467-025-60341-z (PMC12144242; doi:10.1038/s41467-025-60341-z)
Supplement: Supplementary file 2 — Description of Additional Supplementary Files [file 41467_2025_60341_MOESM2_ESM.pdf]

Description of Additional Supplementary Files

Supplementary Video 1 | Cyclic bending test of pristine fallen leaves, white leaves, and LEHs. This video demonstrates the brittleness and fragility of pristine fallen leaves. The white leaves and LEHs demonstrate flexibility and improved mechanical strength. No performance reduction and apparent cracks are observed, meeting the mechanical demands of diverse application scenarios.

Supplementary Video 2 | Powering a calculator with 4 LEHs in series connection. This video demonstrates the capability of using LEHs to power small electronics.

Supplementary Video 3 | Continuously powering a digital clock with 3 LEHs in a series connection. This video demonstrates the continuous power output of LEHs due to the self-regeneration effect, guaranteeing the long-term usage of small appliances.

Supplementary Video 4 | Directly power output of LEH by lighting up an LED. 3 LEHs in a series connection hold sufficient electricity for a red LED with output power in the range of 1-100 microwatts.

Supplementary Video 5 | Power panel through the integration of 24-36 LEHs on a flexible substrate. 24-36 LEHs are in series connection to generate ~10-13 V voltage output, demonstrating the scalability of LEHs.

Supplementary Video 6 | Bending and twisting of the integrated flexible power panel. No performance reduction on the flexible power panel is observed.
